# *N* ^*α*^-acetyl-L-ornithine deacetylase from *Escherichia coli* and a ninhydrin-based assay to enable inhibitor identification

**DOI:** 10.3389/fchem.2024.1415644

**Published:** 2024-07-11

**Authors:** Emma H. Kelley, Jerzy Osipiuk, Malgorzata Korbas, Michael Endres, Alayna Bland, Victoria Ehrman, Andrzej Joachimiak, Kenneth W. Olsen, Daniel P. Becker

**Affiliations:** ^1^ Department of Chemistry and Biochemistry, Loyola University Chicago, Chicago, IL, United States; ^2^ Structural Biology Center, Argonne National Laboratory, X-ray Science Division, Lemont, IL, United States; ^3^ eBERlight, Argonne National Laboratory, X-ray Science Division, Lemont, IL, United States; ^4^ Center for Structural Biology of Infectious Diseases, Consortium for Advanced Science and Engineering, University of Chicago, Chicago, IL, United States; ^5^ Canadian Light Source, Saskatoon, SK, Canada; ^6^ Department of Biochemistry and Molecular Biology, University of Chicago, Chicago, IL, United States

**Keywords:** ArgE, ninhydrin, *Escherichia coli*, enzyme inhibition, X-ray crystal structure

## Abstract

Bacteria are becoming increasingly resistant to antibiotics, therefore there is an urgent need for new classes of antibiotics to fight antibiotic resistance. Mammals do not express *N* ^ɑ^ -acetyl-L-ornithine deacetylase (ArgE), an enzyme that is critical for bacterial survival and growth, thus ArgE represents a promising new antibiotic drug target, as inhibitors would not suffer from mechanism-based toxicity. A new ninhydrin-based assay was designed and validated that included the synthesis of the substrate analog *N* ^5^, *N* ^5^-di-methyl *N* ^α^-acetyl-L-ornithine (k_cat_/K_m_ = 7.32 ± 0.94 × 10^4^ M^−1^s^−1^). This new assay enabled the screening of potential inhibitors that absorb in the UV region, and thus is superior to the established 214 nm assay. Using this new ninhydrin-based assay, captopril was confirmed as an ArgE inhibitor (IC_50_ = 58.7 μM; K_i_ = 37.1 ± 0.85 μM), and a number of phenylboronic acid derivatives were identified as inhibitors, including 4-(diethylamino)phenylboronic acid (IC_50_ = 50.1 μM). Selected inhibitors were also tested in a thermal shift assay with ArgE using SYPRO Orange dye against *Escherichia coli* ArgE to observe the stability of the enzyme in the presence of inhibitors (captopril K_i_ = 35.9 ± 5.1 μM). The active site structure of di-Zn *Ec*ArgE was confirmed using X-ray absorption spectroscopy, and we reported two X-ray crystal structures of *E. coli* ArgE. In summary, we describe the development of a new ninhydrin-based assay for ArgE, the identification of captopril and phenylboronic acids as ArgE inhibitors, thermal shift studies with ArgE + captopril, and the first two published crystal structures of ArgE (mono-Zn and di-Zn).

## Graphical Abstract

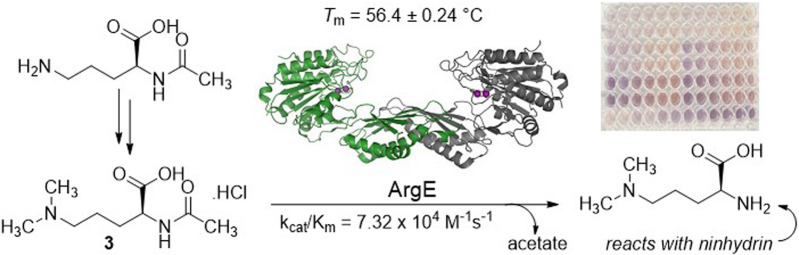

## Introduction

Antibiotic resistance is a grave concern both in the United States and globally. Since the beginning of the COVID-19 pandemic, attention to the problem of antibiotic resistance has diminished and the problem has become even more acute. For example, the CDC reported that in the first year of the pandemic, approximately 30,000 people in the U.S. died from antimicrobial infections, and 40% of these infections were contracted in a hospital setting (Newsroom, 2022). In 2021, the CDC reported some *E. coli* infections as resistant to 9.4% of antimicrobials and listed some strains of *E. coli* as MDR (Center for Disease Control, 2021). This is cause for alarm because antibiotic resistance can be transmitted via horizontal gene transfer (Poirel et al., 2018) allowing a resistant *E. coli* to transfer resistance genes to similar organisms (Keeling and Palmer, 2008). *E. coli* bacterial infections are a primary cause of urinary tract infections (UTIs), bloodstream infections, and pneumonia (Center for Disease Control, 2021), all of which can lead to death. Strategies to address antibiotic resistance include the identification of new antibiotic targets, and one promising bacterial enzyme target is *N* ^*α*^-acetyl-L-ornithine deacetylase (ArgE).

**FIGURE 1:**
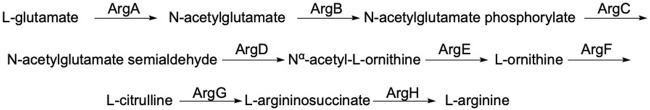
Linear arginine biosynthetic pathway starting with the acetylation of L-glutamate by acetyl-CoA including the deacetylation of N^α^-acetyl-L-ornithine by ArgE and ultimately yielding L-arginine.

**SCHEME 1:**
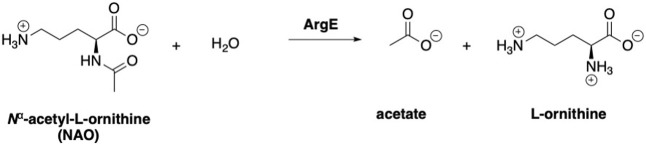
Enzymatic reaction to form acetate and L-ornithine from *N* ^α^-acetyl-L-ornithine (NAO).

ArgE is a metallohydrolase in the arginine biosynthetic pathway (Figure 1) (Javid-Majd and Blanchard, 2000; Ginesy et al., 2015; Sikdar and Kim, 2014) that hydrolyzes *N* ^α^-acetyl-L-ornithine to form acetate and L-ornithine (Scheme 1) (Hlaváček et al., 2014). ArgE is present in all Gram-negative and in the majority of Gram-positive bacteria. Although humans do make arginine, mammalian arginine synthesis differs in two steps that are critical for bacteria to synthesize arginine. The first critical step in bacteria is the acetylation of glutamate to form N-acetyl glutamate, and the second critical and unique step is the deacetylation of *N* ^α^-acetyl-L-ornithine (NAO) by ArgE to form L-ornithine (Sikdar and Kim, 2014). L-Ornithine is critical not only for the synthesis of arginine in bacteria (Margolis et al., 2023), but also for polyamine synthesis, which is required for DNA replication and cell division, making NAO critical for bacterial growth (McGregor et al., 2005; McGregor et al., 2007; Hlaváček et al., 2010; Tao et al., 2012). When Meinnel et al. (1992) transformed an arginine bacterial strain that did not have ArgE with a plasmid containing the argE-gene, an Arg^+^ phenotype was produced. When the start codon (ATG) of the argE-gene was changed to the Amber codon (TAG) in the same plasmid, the plasmid was not able to stop the arginine auxotroph in the same cell strain. Therefore ArgE is required for cell viability, making it an attractive antibiotic target (Meinnel et al., 1992). Because humans do not express ArgE and do not use *N* ^α^-acetyl-L-ornithine to form arginine, inhibitors of ArgE avoid mechanism-based toxicity.

The Holz group (Hlaváček et al., 2010) synthesized and tested 18 ornithine derivatives and conducted MIC tests against *E. coli* and *B. subtilis*. Of the 18 analogs tested, 5 ornithine derivatives were found to be weak inhibitors (200–500 μM) of *B. subtilis* and were therefore able to cross the bacterial cell wall and function as antibiotics, although no antibiotic activity against *E. coli* was found among these inhibitors (Hlaváček et al., 2010). The correlation of *in vitro* inhibitory potency with ArgE enzyme inhibition is consistent with ArgE functioning as an antibiotic target further validating ArgE as an attractive target.

We have been interested in ArgE as a potential antibiotic target in part due to its homology to DapE, which has a similar homodimeric structure and is an enzyme that we have researched extensively for assay development (Heath et al., 2018), structure and mechanism (Nocek et al., 2018; Kochert et al., 2021), alternate substrate and assay optimization (Liveris et al., 2023), and identification of inhibitors (Reidl et al., 2020; DiPuma et al., 2023). Similar to ArgE, DapE is a di-zinc homodimeric hydrolase. DapE hydrolyzes the substrate *N*-succinyl-L,L-diaminopimelic acid releasing succinate and providing L,L-diaminopimelic acid, a precursor to lysine which is also responsible for bacterial cell wall synthesis. DapE and ArgE have similar active site residues, suggesting that a similar series of inhibitors may inhibit both enzymes. The close structural relationship between ArgE and DapE has been previously recognized (Boyen et al., 1992; Meinnel et al., 1992), and they share a similar origin as indicated by their homology (Supplementary Figure S1) (Gouet et al., 2003). An additional link between DapE and ArgE is provided by DapC and ArgD. Both of these enzymes can do the transamination reaction that provides the immediate precursor of DapE. ArgD (Ledwidge and Blanchard, 1999; Charlier and Bervoets, 2019) is the immediate precursor of ArgE, demonstrating that the two pathways and enzymes are inherently very similar and providing an example of enzyme recruitment (Schulenburg and Miller, 2014).

The only reported assay for ArgE inhibition monitors the rate of amide cleavage of the natural substrate of ArgE, *N* ^α^-acetyl-L-ornithine (NAO), at 214 nm (Javid-Majd and Blanchard, 2000). Although this assay is technically simple and easy to perform, it does not allow for complex inhibitors that absorb in the UV region. Therefore, we sought a new assay that takes advantage of the primary amine revealed when ArgE cleaves its substrate. Ninhydrin reacts with primary amines to form a Schiff Base, Ruhemann’s purple, as previously used in a ninhydrin-based assay for DapE enzyme activity (Heath et al., 2018; Liveris et al., 2023). Here we describe the development of a new ninhydrin-based assay for ArgE, the identification of captopril and a series of phenylboronic acids as inhibitors, thermal shift studies of ArgE + captopril, and the first two published crystal structures of ArgE: a mono-zinc structure at 2.13 Å resolution (PDB 7RSF), and a di-zinc structure at 1.8 Å resolution (PDB 8UW6).

## Experimental

### Protein purification

The open reading frame (ORF) of the full-length ArgE protein from *E. coli* was amplified from genomic DNA by PCR and cloned into the pMCSG68 vector. *E. coli* cells harboring the expression plasmid were cultured in LB medium supplemented with ampicillin (100 μg/mL) at 37°C. When the optical density reached 0.8 at 600 nm, the cultures were transferred to 4°C for 1 h. Isopropyl β-D-1-thiogalactopyranoside (IPTG) was added to a final concentration of 0.4 mM for overnight induction at 18°C. Cells were harvested by centrifugation, and disrupted by sonication, and the insoluble cellular material was removed by centrifugation. The ArgE protein was purified using Ni-NTA (Qiagen) affinity chromatography with the addition of 5 mM β-mercaptoethanol in all buffers. The protein was digested with 0.15 mg TEV protease per 20 mg of purified protein for 16 h at 4°C, and then passed through a Ni-NTA column to remove both the TEV protease and cleaved N-terminal tags. The final purification step was size-exclusion chromatography on a HiLoad 16/60 Superdex 200 pg column (GE Healthcare) in 25 mM HEPES buffer pH 7.6, 150 mM NaCl, and 1 mM TCEP. The protein was concentrated on Amicon Ultracel 30K centrifugal filters (Millipore) to a concentration of 20 mg/mL.

### Sequence comparisons

The non-repetitive sequence database was searched for homologs of *Ec*ArgE using the blastp algorithm (Altschul et al., 1990). The sequences for the five DapE proteins and one *Ec*ArgE found were aligned using the Clustal Omega algorithm (Sievers and Higgins, 2002).

### Metal removal from the active site

Apo-*Ec*ArgE enzyme was prepared by extensive dialysis for 3–4 days against 10 mM EDTA in 50 mM HEPES, pH 7.5 (Gillner et al., 2009).

### Metal insertion at the active site

Apo-*Ec*ArgE was extensively dialyzed against a metal-free 50 mM HEPES buffer, pH 7.5. ArgE was then extensively dialyzed against 0.1 mM ZnCl_2_ (99.999%; Strem Chemicals, Newburyport, MA, United States) in 50 mM HEPES buffer, pH 7.5 followed by exhaustive dialysis against a metal-free 50 mM HEPES buffer, pH 7.5. The Zn-inserted enzyme was then utilized in enzymatic assays.

### Buffer switch to KP_i_

The reconstituted ArgE enzyme in 50 mM HEPES buffer, pH 7.5 was extensively dialyzed against 50 mM KP_i_, pH 7.5. The Zn-inserted enzyme (KP_i_ buffer, pH 7.5) was utilized in enzymatic assays.

### Synthesis of di-methyl N^α^-acetyl-L-ornithine

*N* ^*α*^ *-*Acetyl-L-ornithine (NAO, **1**, 99 mg, 0.57 mmol) and formaldehyde (2.85 mmol) were stirred for 15 minutes in 2% methanol and 98% acetonitrile (0.2 M) under argon, then sodium cyanoborohydride was added and the reaction was stirred under argon for 15 minutes. Acetic acid (1.14 mmol) was then added and the mixture was stirred under argon at room temperature until the starting material was consumed as determined by thin layer chromatography. The solution was dried over sodium sulfate and evaporated under reduced pressure. The residue was then crystallized from 2-propanol, which was exposed to diethyl ether in a two-chamber solvent system. The zwitterionic internal salt **2** was then converted to the HCl salt **3** by the addition of 3.5 equivalents of 2.0 M HCl in ether. The resulting solid was collected and crystallized from 2-propanol exposed to diethyl ether in a two-chamber recrystallization system resulting in di-methyl-N^*α*^-acetyl-L-ornithine **3** as a white, crystalline solid (95.2 mg, 70%), mp 198.2°C–200°C (dec.). ^1^H NMR (500 MHz, D_2_O, doubling of some peaks due to amide rotamers) δ 4.35–4.25 (m, 1H), 3.12 (dd, J = 9.6, 6.0 Hz, 1.57H), 3.02–2.93 (m, 0.34H), 2.82 (d, J = 1.8 Hz, 4.57H), 2.59 (s, 0.50H), 2.19–2.12 (m, 0.19H), 2.11–2.04 (m, 0.25H), 1.99 (d, J = 1.2 Hz, 3H), 1.93–1.81 (m, 1.30H), 1.80–1.66 (m, 2.91H), 1.62 (q, J = 7.3 Hz, 0.36H). ^13^C NMR (126 MHz) δ 175.5, 174.26 56.9, 52.4, 48.9, 42.7, 27.5, 21.7.

### 214 nm ArgE enzyme assay protocol

ArgE enzyme activity was measured as described by Javid-Majd and Blanchard (Javid-Majd and Blanchard, 2000) and later by Holz (McGregor et al., 2005), who observed the hydrolysis of *N* ^α^-acetyl-L-ornithine (NAO) or di-methyl-*N* ^α^-acetyl-L-ornithine spectrophotometrically at 25°C by monitoring the peptide bond cleavage by monitoring the decrease in absorbance at 214 nm. Specifically, the continuous assay was performed at 214 nm in a spectrophotometer at 30°C. The volume of each component was adjusted to give a total reaction volume of 1,000 µL. The *N* ^α^-acetyl-L-ornithine was purchased and the di-methyl-*N* ^α^-acetyl-L-ornithine substrate was synthesized as described above. The final concentration of the substrates (NAO and di-Me NAO) was 2 mM in the assay for the screening and IC_50_ experiments and 0 mM–2.5 mM in the kinetic experiments. The final concentration of the *Ec*ArgE enzyme was 10 nM. Potential inhibitors were dissolved in 50 mM KP_i_ buffer and screened at 100 μM in triplicate and at various concentrations for an IC_50_ experiment. The potential inhibitor was added to the 50 mM KP_i_ buffer, pH: 7.5, immediately followed by the ArgE enzyme and incubated at 30°C for 10 min. Following a 10-min incubation time, the substrate was added, and the mixture was pipetted to mix and then pipetted into a quartz cuvette (l = 1 cm) and placed in the spectrophotometer. Measurements were collected in 1-s increments for 300 s (5 min). The data were exported and IC_50_s and kinetic constants were calculated using a non-linear regression Michaelis-Menten equation in GraphPad Prism.

### Enzymatic assay protocol, ninhydrin assay

A discontinuous assay was performed utilizing a Techne PCR Thermal Cycler System and assays were run with a total reaction volume of 100 µL and a final enzyme concentration of 10 nM. Potential inhibitors were dissolved in DMSO, and the pre-assay concentrations were adjusted to give a final concentration of 5% DMSO in the assay. It was observed that DMSO concentrations higher than 5% inhibited the enzyme. The selected inhibitors were dissolved in 50 mM KP_i_ buffer, pH: 7.5 at 30°C and screened at 100 μM or other concentrations, depending on the type of experiment, followed by the *Ec*ArgE enzyme and incubated for 10 min. A 2 mM solution (for screening and IC_50_ experiments) or 0 mM–6.0 mM (for kinetic experiments) of di-methyl-*N* ^α^-acetyl-L-ornithine (**3**) was added and subjected to enzymatic cleavage for 10 min (when running incubation studies the incubation time was 2–10 min in increments of 2 min) followed by heating to 99°C for 1 min and then cooling to 0°C. A 2% ninhydrin solution (50 μL) was added, and the mixture was mixed by pipetting while cooled to 0°C. The reaction was then heated to 100°C for 10 min. The absorbance of an 80 μL aliquot was recorded at 570 nm on a BioTek Synergy 2 microplate reader. The IC_50_ values and kinetic constants were calculated using GraphPad Prism using non-linear regression and the Michaelis-Menten equation.

### Molecular docking protocol

The inhibitor 4-(diethylamino)phenylboronic acid (Entry 4, Table 2) was built in the Molecular Operating Environment (MOE) with both hydroxyl groups of the phenylboronic acid deprotonated. The ligand was optimized at 310 K, pH 7.4 and a salt value of 0.1 using Protonate 3D and energy was minimized using the MMFF94x force field. A database of the ligand was created after the optimization and minimization steps. The X-ray crystal structure of *Ec*DapE (PDB 8UW6) was uploaded into MOE and prepared for docking following MOE’s Structure Preparation utility. The hydrogen-bonding network of the docking active site was further optimized at 310 K, pH: 7.4, and a salt value of 0.1 using Protonate 3D. The substrate binding pocket was cleared of ligands including Tris. Following the preparation of the small molecule ligands and the *Ec*ArgE docking receptor model, an induced-fit molecular docking was carried out with the entire receptor (enzyme active site) using the ligand database. The alpha triangle placement method with affinity dG scoring generated 300 poses, which were further refined using the induced fit method with GBVI/WSA dG scoring to obtain the top 100 poses. The Amber14:EHT force field was used to perform these calculations.

### Molecular dynamics simulations to identify fixed waters in ArgE

Starting with the X-ray structure (PDB 8UW6) of the AB dimer, a combination of energy minimization and molecular dynamics was used to determine which water molecules were tightly bound to the protein. Each simulation box, containing one dimer, a TIP3 water box extending at least 10 Å beyond the protein in all directions, and 0.1 M NaCl adjusted to neutralize the charge in the water box, was assembled using the molecular graphics program VMD (Humphrey et al., 1996). The simulation box was then brought to equilibrium using the molecular dynamics program NAMD (Phillips et al., 2005). The equilibration procedure involved energy minimization with and without restraints on the protein coordinates (3,000 steps each), slow heating from 10 to 310 K (30,000 steps), and then pressure equilibration using a Langevin piston (10,000 steps) followed by unrestrained dynamics for 5,000,000 steps. The time step was 2 fs with every 150th step saved in the trajectory for analysis. Periodic boundary conditions were used. The cutoffs for non-bonding (van der Waals and electrostatic) interactions were 15 Å. The switch distance was 13 Å, and a 1.01 ± 4 scaling factor was used. All calculations were done using CHARMM 36 parameters (Huang et al., 2017). (molecular graphics diagrams were generated using VMD (Humphrey et al., 1996).

### Thermal shift assay protocol

For thermal shift assays, *Ec*ArgE was used at a final concentration of 5 μM and SYPRO Orange was used at a final concentration of 10X (purchased as a 5000X concentrate in DMSO, equivalent to a 10 mM solution (Steinberg, 2009) from Thermo Fisher Scientific), and experiments were performed on a Step One Real-Time PCR System™ (Thermo Fisher Scientific). The experiment was carried out in 10 µL triplicates in 50 mM KP_i_ buffer at pH: 7.5, nanopure water, and the inhibitor concentrations examined were based on the IC_50_ of the inhibitors. Sample solutions were dispensed into a 96-well optical reaction plate (Thermo Fisher Scientific) and the plate was sealed with an optical PCR plate sheet (Thermo Fisher Scientific). The temperature was continuously increased at a ramp rate of 0.05°C/sec. for 2 min at 25°C and then increased at a ramp rate of 0.05°C/s for 2 min at 99°C. Data were collected every 0.4°C. Melting curves were obtained from the negative derivative and exported from the instrument to Microsoft Excel. Fluorescence data were analyzed using a Savitzky-Golay algorithm and differentiated to the third derivative. Melting temperatures (T_m_) were plotted against the log of the concentration, and the K_i_s were calculated using a derived Van’t Hoff equation (Bhayani and Ballicora, 2022).

### X-ray absorption spectroscopy (XAS) protocol

To confirm the success of zinc reconstitution and to establish a protocol for future X-ray Absorption Spectroscopy studies, we conducted XAS studies on apo and di-Zn *Ec*ArgE. X-ray absorption spectroscopy data were collected in November 2022 at the Advanced Photon Source (APS), Beamline 20-BM. The enzyme was in liquid solution form and prior to being placed in the chamber, it was frozen in liquid nitrogen. The experiment was conducted at 78 K using liquid nitrogen as a cryostat. To conduct the experiment and to prevent cumulative X-ray radiation damage, we chose six locations within the sample and performed 42 runs (seven runs at each location) to obtain a Zn count of approximately 1 million. The raw data were entered into the XAS data analysis suite Larch (Newville, 2013) for further data processing. The data were inspected, and outliers were removed, for example, due to sample deterioration or if the spot chosen was covered with ice crystals. Each XAS spectrum was then calibrated to a Zn-foil K-edge energy of 9659 eV (Booklet, 2001). All calibrated XAS spectra were then merged. The extracted k^3^-weighted EXAFS spectrum (k_min_ = 2.5 Å^-1^ and k_max_ = 13.5 Å^-1^) was fitted in Larch and the initial FEFF input file (required to calculate the scattering path parameters) was made using Zn451 (PDB 3PFO) as the origin (0,0,0). To fit the data, we initially considered only single scattering paths to determine the first shell composition (Supplementary Tables S1, S2). To obtain the final fit, we also considered multiple scattering paths within the imidazole ring of the histidine residue, with the number of legs ≤3, and fixed S_0_^2^ at 1.0, a typical fixed value for Zn metals (Costello et al., 2006; Yano and Yachandra, 2009; Gou et al., 2018; Mishra et al., 2020; Naito et al., 2022). Due to the limited resolution of the EXAFS spectrum, the distances to the first and second shell O and N atoms and their respective Debye-Waller factors, were refined together. In order not to over-interpret the analyzed EXAFS spectrum, we also collectively refined the Debye-Waller factors for both single- and multiple-leg scattering paths within the imidazole ring. In addition, the imidazole ring was treated as a rigid structure and only the Zn-N distance was allowed to vary freely during the refinement.

### Protein crystallization

The ArgE protein was crystallized using vapor diffusion in sitting droplets. A 0.4 µL aliquot of protein was mixed with a 0.4 µL of crystallization reagent and allowed to equilibrate over 145 µL of crystallization reagent in CrystalQuick 96-well Greiner plates (Hampton Research). Pipetting was performed using a Mosquito nanoliter liquid handling system (TTP LabTech). The MCSG crystallization suite (Microlytic) was employed for four screens, and Pi-minimal screens (Jena Bioscience)(Gorrec et al., 2011) were used for crystallization trials at 16°C. The best crystals of ArgE protein in mono-Zn form were obtained from the C7 conditions of the MCSG3 screen (1 M ammonium citrate, 0.1 M Bis-Tris propane pH 7.0) after 7 months of incubation. The best crystals of the di-Zn form of ArgE protein were obtained from E4 conditions of the MCSG1 screen (0.2 M lithium sulfate, 30% PEG 3350, 0.1 M Tris buffer pH 8.5) after 2 months of incubation. Crystals of mono- and di-zinc proteins were briefly soaked in crystallization buffers supplemented with either 25% glycerol or 15% ethylene glycol, respectively, as cryo-protectants and then flash-cooled in liquid nitrogen.

### Data collection, structure determination and refinement

Single-wavelength X-ray diffraction data were collected at 100 K temperature at the 19-ID beamline of the Structural Biology Center (Rosenbaum et al., 2006) at the Advanced Photon Source at Argonne National Laboratory for the mono-zinc protein crystals and at the 17-ID-2 (FMX) beamline (Schneider et al., 2021) of the National Synchrotron Light Source II at Brookhaven National Laboratory for the di-zinc protein crystals. The intensities were integrated and scaled with the HKL3000 suite (Minor et al., 2006). The initial ArgE protein structure was determined by single-wavelength anomalous diffraction (SAD) phasing using zinc anomalous scattering and the HKL3000 suite, which includes the programs SHELXC, SHELXD, SHELXE (Schneider and Sheldrick, 2002), MLPHARE, and SOLVE/RESOLVE (Terwilliger, 2003). The initial structure was used to solve subsequent structures by the molecular replacement method using the HKL3000 suite with the MOLREP program (Vagin and Teplyakov, 2010). Several rounds of manual adjustments of models using COOT (Emsley and Cowtan, 2004) and refinement with the Refmac program (Murshudov et al., 1997) from the CCP4 suite (Project, 1994) were performed to obtain the final structures. The position and occupancy of the zinc ions were assigned based on anomalous and fo-fc difference electron density maps. The stereochemistry of the structure was validated with the PHENIX suite (Adams et al., 2002) incorporating MOLPROBITY tools (Davis et al., 2004). A summary of the data collection and refinement statistics is given in Supplementary Table S3 (Davis et al., 2004; Karplus and Diederichs, 2012).

### Coordinates

The atomic coordinates and structure factors of the ArgE protein structures were deposited into the Protein Data Bank as 7RSF and 8UW6.

### Molecular dynamics protocol for zinc insertion

The *Ec*ArgE mono-zinc (PDB 7RSF) with a second zinc built into the model using the coordinates from *Hi*DapE (PDB 5UEJ) was used as the structural model for molecular dynamics. For all molecular dynamics experiments, a simulation box was created using the molecular graphics program VMD(Humphrey et al., 1996). The water box extended an additional 10 Å from the edge of the protein and contained 0.1 M NaCl. The simulation box was brought to equilibrium using the molecular dynamics program NAMD (Phillips et al., 2005). The equilibration procedure was the same as described above for the molecular dynamics of fixed water molecules.

### Superimposing DapE structures

The Multiseq routine (Roberts et al., 2006) in the VMD graphics program (Humphrey et al., 1996) was used to superimpose domains in different ArgE structures to evaluate the flexibility of the hinge region of the protein.

## Results and discussion

### Synthesis of the di-methyl N^α^-acetyl-L-ornithine alternate substrate

Previously, Blanchard developed a simple, continuous 214 nm assay (Javid-Majd and Blanchard, 2000) using the endogenous substrate NAO to test potential inhibitors of ArgE. The 214 nm assay is a simple and reproducible assay but it is not useful for inhibitors that absorb strongly in the UV region, therefore excluding many drug-like molecules, as high background UV absorption would lead to greater background and obscure the actual enzyme cleavage data. Furthermore, the 214 nm assay is limited to buffers that do not absorb strongly at 214 nm, which compromises the ability to dissolve many potential inhibitors. As an alternative assay, we explored another potential substrate, p-nitrophenyl acetate, in the hope that ArgE would cleave the acetate to yield the product p-nitrophenol, but we observed no cleavage based on monitoring at 405 nm (Means and Bender, 1975). We also attempted the use of the DapE alternate substrate *N* ^6^-methyl-*N*-succinyl-L,L-diaminopimelic acid (*N* ^6^-methyl-SDAP) (Heath et al., 2018) because of the similarities between the ArgE and DapE active site structure but observed no cleavage of this synthetic substrate at 225 nm. We therefore designed a ninhydrin-based assay similar to our DapE ninhydrin-based assay (Heath et al., 2018) to enable the analysis of more structurally diverse inhibitors.

**SCHEME 2:**
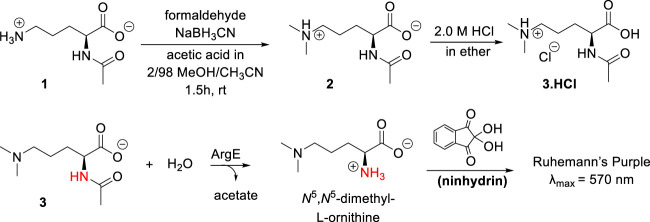
Synthesis of *N* ^5^,*N* ^5^-dimethyl-*N* ^α^-acetyl-L-ornithine, and formation of Ruhemann’s purple via acetyl cleavage of *N* ^5^,*N* ^5^-dimethyl *N* ^α^-acetyl-L-ornithine **3** by ArgE.

When ArgE cleaves NAO, it forms L-ornithine and acetate (Scheme 1). L-Ornithine has two primary amines, both of which would react with ninhydrin, therefore we needed to synthesize a mono- or di-methylated substrate to utilize ninhydrin in an assay to detect only the L-ornithine product of hydrolysis. Based on our recently reported results with the *N* ^6^,*N* ^6^-dimethyl-L,L-SDAP substrate for DapE (Liveris et al., 2023), we focused on the synthesis of the *N* ^5^,*N* ^5^-dimethyl-L-ornithine ArgE alternate substrate **3** (Scheme 2).

The synthesis of the *N* ^5^,*N* ^5^-dimethyl-L-ornithine substrate was completed as shown in Scheme 2. *N* ^α^-acetyl-L-ornithine (**1**) was subjected to reductive amination (Mitchell and Finney, 2000) to form the *N* ^5^,*N* ^5^-dimethyl zwitterionic salt (**2**). From this crystalline internal salt, the HCl salt **3** was prepared using 2.0 M HCl in diethyl ether, which was crystallized from isopropyl alcohol by subjecting it to the slow addition of diethyl ether in a vapor diffusion chamber. With pure *N* ^5^,*N* ^5^-di-methyl *N* ^α^-acetyl-L-ornithine hydrochloride (**3**) in hand, the ninhydrin-based ArgE assay was then validated.

### Development of the ninhydrin assay and identification of inhibitors of EcArgE

As discussed earlier, ninhydrin reacts with free amines to form a Schiff Base, Ruhemann’s purple (Scheme 2). (Heath et al., 2018; Liveris et al., 2023) Encouraged by the homology between DapE and ArgE, we created a ninhydrin-based assay for ArgE. *N* ^5^,*N* ^5^-dimethyl *N* ^α^-acetyl-L-ornithine hydrochloride substrate **3** was tested against *Ec*ArgE to confirm if it functions as a substrate, and we observed cleavage of di-methyl *N* ^α^-acetyl-L-ornithine (**3**) to form Ruhemann’s purple. To confirm that the 10-min incubation time of substrate + enzyme is measuring a constant initial velocity, we ran kinetic assays with 2-min to 10-min incubation times. We observed that at each substrate concentration, varying the incubation time resulted in a linear response with *R* ^2^ values all ≥0.88 (Supplementary Figure S7).

We then conducted kinetic assays to obtain the k_cat_/K_m_ value to compare the k_cat_/K_m_ value with the ninhydrin assay *versus* the 214 nm assay and the natural substrate, *N* ^*α*^-acetyl-L-ornithine (NAO), to ensure that the new substrate was working at the same relative rate as the endogenous substrate. The k_cat_/K_m_ value for the di-methylated substrate (Table 1) and the endogenous substrate **1** (Table 1) are statistically identical (Supplementary Figure S8), indicating that the *N* ^5^,*N* ^5^-dimethyl-*N* ^α^-acetyl-L-ornithine hydrochloride alternate substrate **3** is cleaved at the same rate as the endogenous substrate. The k_cat_/K_m_ for the di-methylated substrate **3** (7.32 × 10^4^ s^−1^/M^−1^) in the ninhydrin-based assay is statistically the same as in the 214 nm assay (8.55 × 10^4^ s^−1^/M^−1^).

**TABLE 1:** Comparison of the inhibitory potency of captopril as determined by the ninhydrin assay, the 214 nm assay, and TSA to validate the new ninhydrin-based assay with *N* ^5^,*N* ^5^-dimethyl *N* ^α^-acetyl-L-ornithine (**3**) as the substrate.

Experiment	Ninhydrin assay	214 nm assay	214 nm assay	TSA
Substrate	**3**	**3**	NAO	-
IC_50_ (μM) of captopril	59.1 ± 8.1	67.1 ± 5.1	-	-
K_i_ (μM)	37.1 ± 0.85	-	-	35.9 ± 5.1
k_cat_/K_m_	7.32 ± 0.94 × 10^4^ s^-1^/M	8.55 ± 0.98 × 10^4^ s^-1^/M	9.86 ± 0.96 × 10^4^ s^-1^/M	-

Assay conditions were determined to be optimal using 2 mM substrate and 10 nM *Ec*ArgE. To validate the ninhydrin assay, parallel experiments were performed with the 214 nm assay with the same substrate and enzyme concentration (Table 1). We determined that the IC_50_ value for captopril is 59.1 ± 8.1 μM for the ninhydrin assay (Supplementary Figure S9A) and 67.1 ± 5.1 μM as determined by the 214 nm assay (Supplementary Figure S9B), which are statistically identical, validating the new ninhydrin assay against the previously reported 214 nm assay (Javid-Majd and Blanchard, 2000). Kinetic experiments were performed with the ninhydrin assay and with a thermal shift assay. Using captopril as an inhibitor, the K_i_ values from the ninhydrin assay (K_i_ = 37.1 ± 0.85 μM, Supplementary Figure S10) and the thermal shift assay (K_i_ = 35.9 ± 5.1 μM, Supplementary Figure S14) were statistically the same, providing further validation of the ninhydrin assay.

With initial validation studies complete, potential inhibitors of *Ec*ArgE were screened. We employed the new ninhydrin-based assay using the synthetic substrate analog, *N* ^5^,*N* ^5^-dimethyl-*N* ^α^-acetyl-L-ornithine (**3**) to screen potential inhibitors. Captopril was selected for testing as a potential inhibitor of ArgE, as it is the most potent inhibitor reported for DapE (IC_50_ = 3.3 μM; K_i_ = 1.82 ± 0.09 μM, competitive)(Gillner et al., 2009), and a co-crystal structure of DapE with captopril has been reported (Starus et al., 2015). Captopril was confirmed as an inhibitor of ArgE (Table 2), although it is significantly less potent against ArgE, exhibiting 57.8% inhibition at 100 μM, and an IC_50_ = 58.7 ± 5.8 μM. Captopril is a competitive inhibitor of ArgE, as it is for DapE.

**TABLE 2:** Inhibition of *Ec*ArgE by phenylboronic acids and benzoic acids.

Entry	Structure	MW (g/mol)	clogP^a^	% Inhibition at 100 μM
1	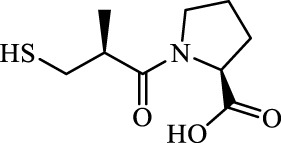	217.3	0.58	57.8 ± 0.04
2	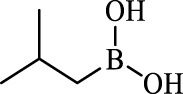	101.9	0.57	11.0 ± 8.7
3	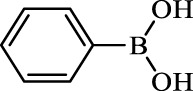	121.9	1.04	41.9 ± 1.8
4	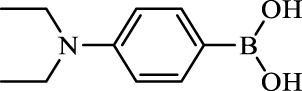	193.1	2.12	91.5 ± 1.5
5	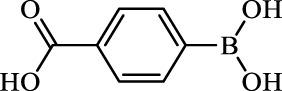	165.9	0.67	80.4 ± 3.7
6	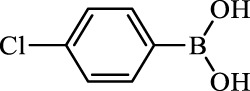	156.4	1.66	68.7 ± 1.2
7	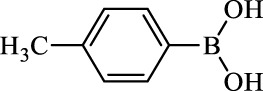	136.0	1.47	59.0 ± 9.4
8	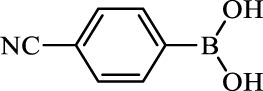	146.9	0.77	48.0 ± 2.6
9	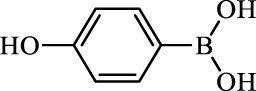	137.9	0.63	29.8 ± 11.2
10	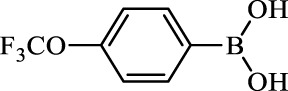	205.9	2.20	25.5 ± 2.4
11	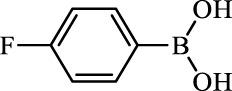	139.9	1.20	8.1 ± 4.1
12	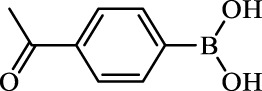	164.0	0.89	15.8 ± 1.9
13	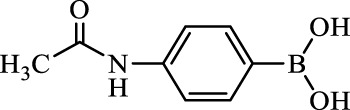	179.0	0.28	24.0 ± 6.9
14	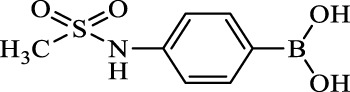	215.0	0.30	23.5 ± 6.9
15	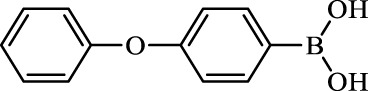	214.0	2.53	48.8 ± 2.7
16	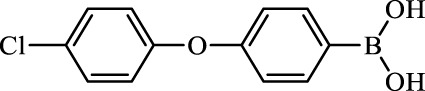	248.5	3.15	55.7 ± 4.9
17	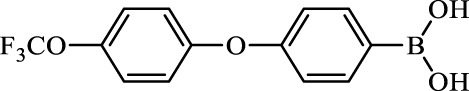	298.0	3.68	36.0 ± 5.8
18	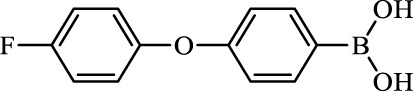	232.0	2.69	9.3 ± 1.8
19	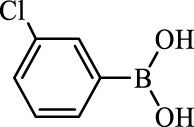	156.4	1.66	19.7 ± 3.0
20	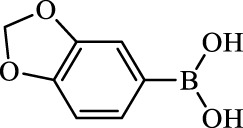	165.9	0.79	47.0 ± 2.7
21	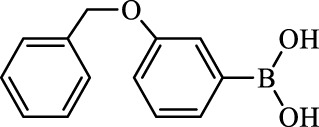	228.1	2.66	40.3 ± 6.8
22	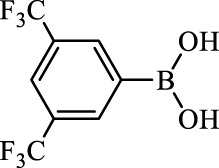	257.9	2.88	0.0 ± 1.8
23	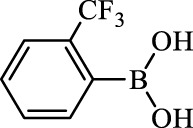	189.9	1.96	40.6 ± 9.0
24	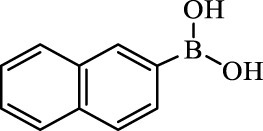	172.0	2.30	16.2 ± 6.1
25	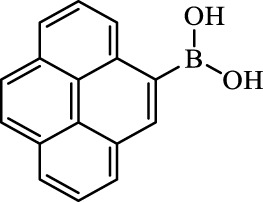	246.1	0.44	15.9 ± 4.0
26	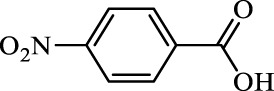	167.1	1.55	57.9 ± 7.1
27	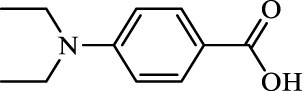	193.2	2.73	53.4 ± 2.6
28	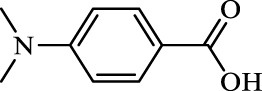	165.2	1.88	41.0 ± 10.8
29	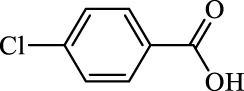	156.6	2.27	28.0 ± 2.3

^a^ clogP is the calculated logP, which is the logarithm of the partition coefficient of a compound's partition coefficient between n-octanol and water.

Boronic acids were also identified as DapE inhibitors (Gillner et al., 2009) and therefore we assayed a number of commercially available boronic acids (Table 2). Isobutylboronic acid (entry 2) was nearly inactive (11% inhibition at 100 μM), but phenylboronic acid (entry 3) inhibited ArgE by 41.9% at 100 μM, suggesting that the phenyl ring may be important for enzyme inhibition. Substitution at the para position led to more potent inhibitors, notably 4-(diethylamino)phenylboronic acid (entry 4, 91.5% inhibition at 100 μM; IC_50_ = 50.1 μM, Supplementary Figure S11) and 4-carboxyphenylboronic acid (entry 5, 80.4% inhibition, IC_50_ = 54.1 μM, Supplementary Figure S12), which were slightly more potent than captopril. The 4-chlorophenylboronic acid (entry 6, 68.7%) was comparable to the 4-methyl derivative (entry 7), but surprisingly more potent than the 4-fluoro derivative (entry 11). The more bulky 4-phenyloxyphenyl derivative (entry 15) inhibited ArgE by 48.8% at 100 μM, and the 4-chloro diaryl ether (entry 16) was somewhat more potent (55.7%), but the corresponding para-fluoro derivative (entry 18) was much less potent (9.3%). The 3-substituted derivatives were moderate inhibitors, led by the methylenedioxy derivative (entry 20) with 47.0% inhibition, while the 3,3-bis-trifluoromethyl derivative (entry 22) lacked activity, presumably due to negative steric interactions. The polycyclic aromatic boronic acids (entries 24 and 25) were notably less potent.

To further explore SAR we tested several commercially available benzoic acids as isosteres of the phenylboronic acids. Carboxylic acids (entries 26–29) were assessed, with the 4-nitrobenzoic acid (entry 26) being the most potent benzoic acid (57.9%), and the 4-diethylaminophenyl analog (entry 27), a direct analog of boronic acid (entry 4), being less potent (53.4%) than the analogous boronic acid (entry 4, 91.5%), showing boronic acids as more potent relative to the corresponding bioisosteric carboxylic acids.

### Molecular docking

**FIGURE 2:**
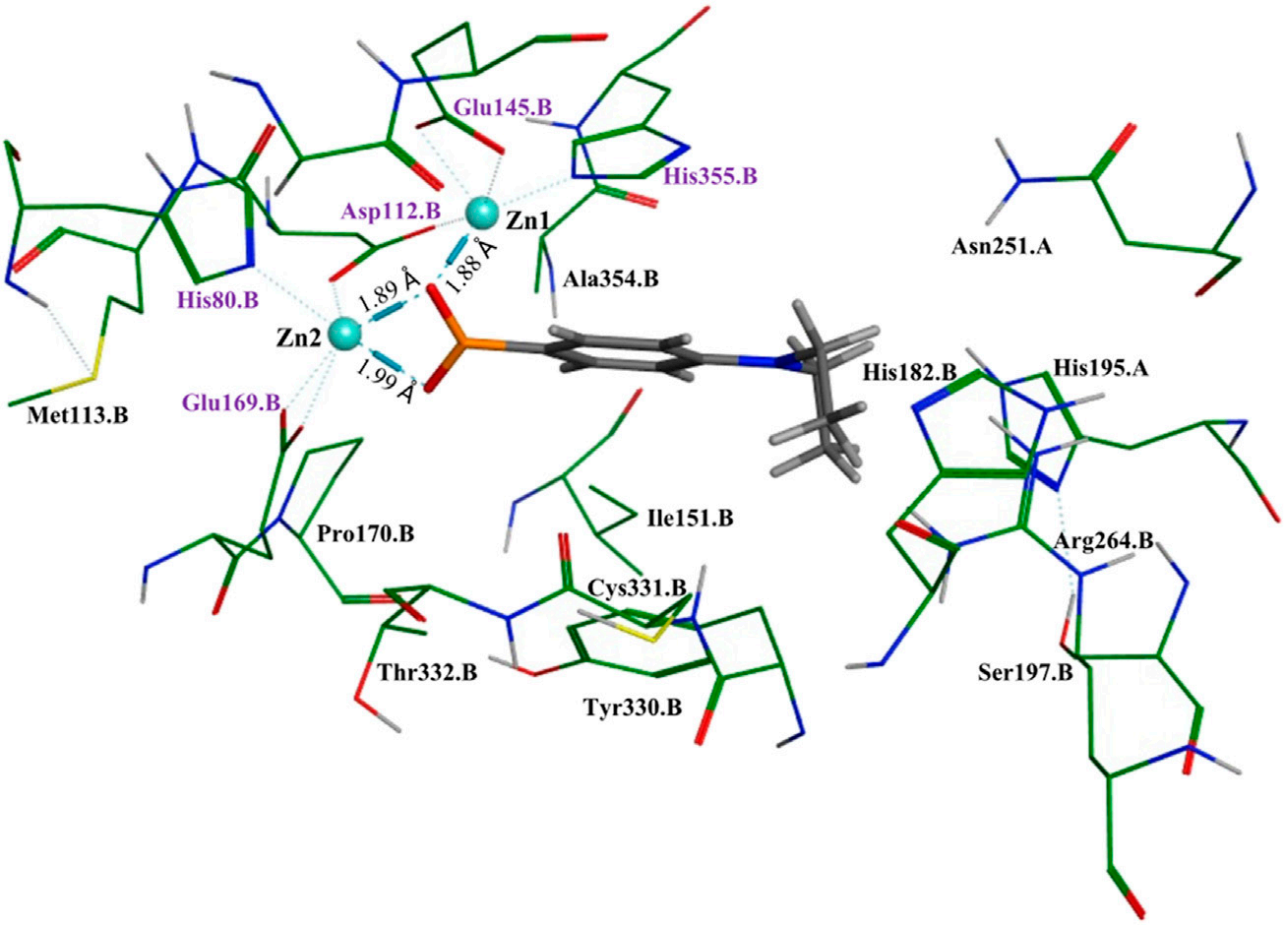
Molecular docking of *Ec*ArgE (PDB 8UW6) with 4-(diethylamino)phenylboronic acid, Table 2, entry **4**. The purple text indicates the zinc-interacting active site residues.

To better understand and visualize how inhibitors may be binding to our enzyme, we ran molecular docking experiments on the most potent *Ec*ArgE inhibitor identified to date, 4-(diethylamino)phenylboronic acid (Table 2, Entry 4) with *Ec*ArgE (PDB 8UW6). It was observed that one of the deprotonated oxygen atoms of the boronic acid forms a bridging interaction with the two zinc atoms (Figure 2). The second deprotonated oxygen of the boronic acid interacts with one of the active site zincs. Our docking results are consistent with the Petsko group’s X-ray crystal structure of the aminopeptidase *Aeromonas proteolytica*, a di-zinc enzyme that contains similar active site amino acid residues as ArgE, bound to 1-butaneboronic acid (De Paola et al., 1999). The diethylamino moiety of this phenylboronic acid derivative extends into the solvent.

### Thermal shift assay of captopril

We performed a thermal shift assay (Bhayani and Ballicora, 2022) with captopril against *Ec*ArgE. Optimization studies indicated that 5 μM enzyme and 10X (equivalent to 20 μM (Steinberg, 2009)) SYPRO Orange dye were the best assay conditions identified. We observed that at the T_m_ (56.4°C) of *Ec*ArgE (Table 3) there was a stabilization of the enzyme when the inhibitor was binding, as demonstrated by the +4°C positive shift in the melting temperature when captopril is bound. This indicates that the inhibitor is binding primarily to the native folded state of the enzyme (Cimmperman et al., 2008; Bhusal et al., 2017). From these data, we were able to calculate a K_i_ value of 35.9 μM (Supplementary Figure S14). Like DapE (Liveris et al., 2023), *Ec*ArgE has two melting temperatures, the first with a positive shift, which is reported in Table 3, and the second with a slight and less consistent decrease (Kelley et al., 2024), which is not reported in the table below. The two melt temperatures suggest two different globule forms of the enzymes. The second melt temperature is not reported because it is not relevant to the native, folded enzyme and the kinetic constant calculations.

**TABLE 3:** Thermal shift results of *Ec*ArgE with captopril and K_i_ calculated from T_m_.

[captopril] (μM)	T_m_^*^ (°C)	K_i__(μM)_
0	56.4 ± 0.24	
1	56.4 ± 0.17	
10	56.6 ± 0.07	
30	56.6 ± 0.11	35.9 ± 5.1
50	57.8 ± 0.30	
70	58.5 ± 0.29	
90	59.1 ± 0.13	
100	60.0 ± 0.35	

*5 μM *Ec*ArgE, 10X Dye.

### X-ray absorption spectroscopy study

**FIGURE 3:**
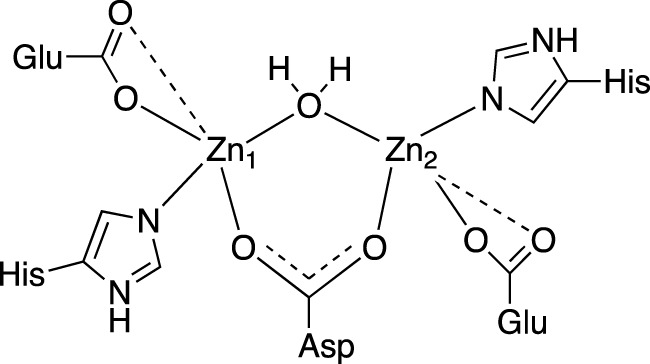
2D active site structure of *Ec*ArgE from XAS data (Tao et al., 2012).

Our analysis of the EXAFS data of *Ec*ArgE reveals the presence of two zinc atoms, and a Zn-Zn distance of 3.39 Å , which is consistent with the crystallographic Zn-Zn distance and with previously reported XAS results. Our proposed active site structure (Figure 3) based on XAS data is consistent with previously reported Zn-Zn *Ec*ArgE data (Tao et al., 2012), X-ray crystallographic data of *Ec*ArgE as well as multiple DapE species (Supplementary Figure S1) (Gouet et al., 2003), an enzyme that is homologous and evolutionarily linked to ArgE (Boyen et al., 1992).

The XAS fitting confirms (Supplementary Tables S1, S2) the success of zinc incorporation by dialysis of *Ec*ArgE and the data are consistent with previous Zn-Zn *Ec*ArgE XAS data (Tao et al., 2012) and X-ray crystallography confirming that *Ec*ArgE is a di-Zn metalloenzyme. With this confirmation, even before obtaining crystal structures, we proceeded with enzymatic assays using fully occupied di-zinc *Ec*ArgE.

### Structural comparison of EcArgE with other ArgE structures and DapE

**FIGURE 4:**
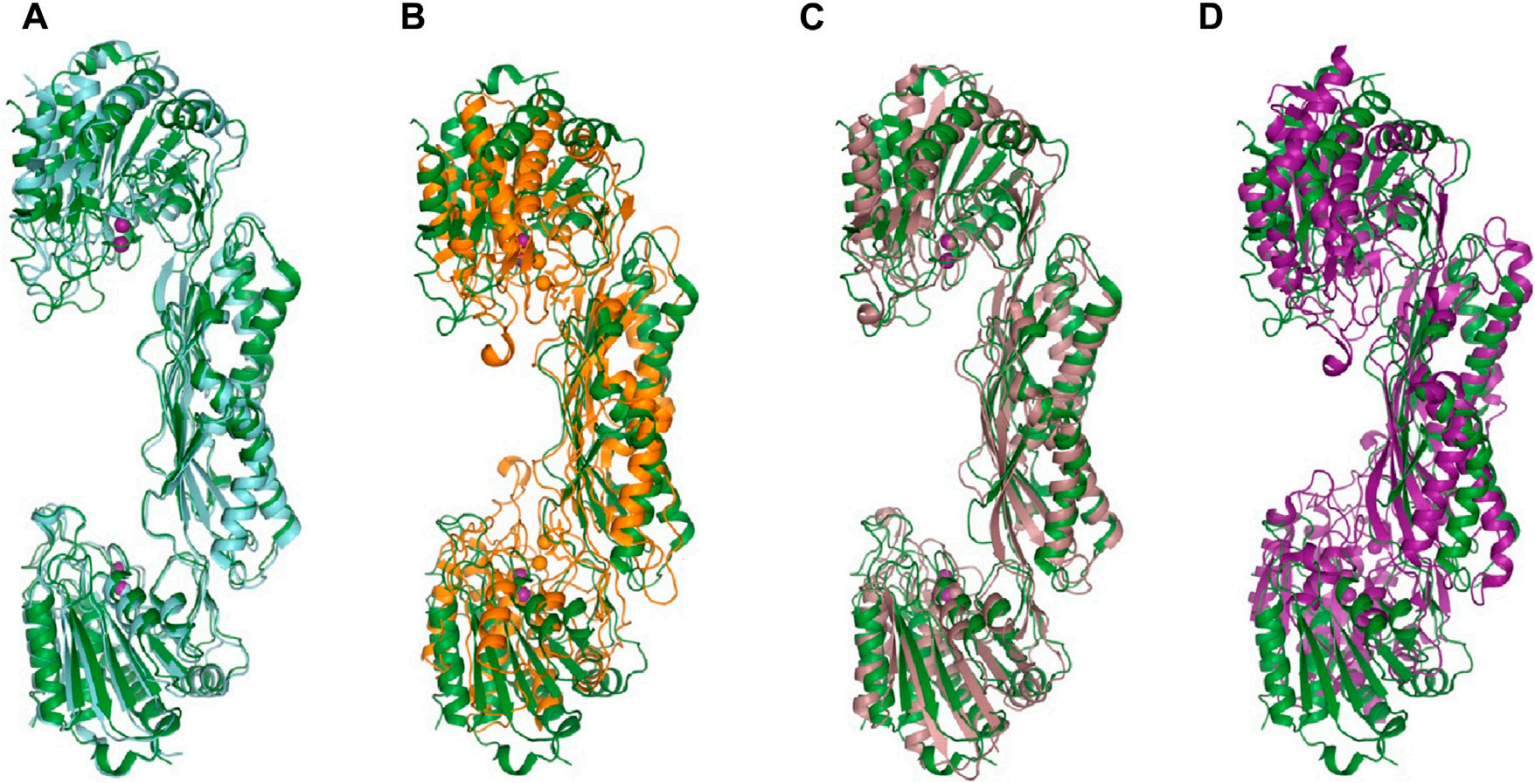
The structure of *E. coli* ArgE di-zinc form (green) is compared to similar structures. **(A)** *Ec*ArgE mono-zinc form (PDB 7RSF) (cyan), **(B)** *Haemophilus influenzae* DapE (PDB 5VO3) (orange), **(C)** *Enterococcus faecium* DapE (PDB 7UOI) (brown), **(D)** *Rhodopseudomonas palustris* ArgE (PDB 3PFO) (purple).

ArgE hydrolyzes its endogenous substrate *N* ^α^-acetyl-L-ornithine (NAO) yielding L-ornithine and acetate. To further understand the structure of the active site in order to gain mechanistic insight into the enzymatic reaction, we obtained structures of *Ec*ArgE protein in two forms: mono-zinc at 2.13 Å resolution (PDB 7RSF), and di-zinc at 1.8 Å resolution (PDB 8UW6). Protein chains were modeled for both structures with the exception of three N-terminal residues. The obtained *Ec*ArgE structures closely resemble known structures of DapE and ArgE/DapE-related proteins from the M20 metallopeptidase family (Figure 4; Supplementary Figure S1) (Shi et al., 2007; Nocek et al., 2010; Nocek et al., 2018).

**FIGURE 5:**
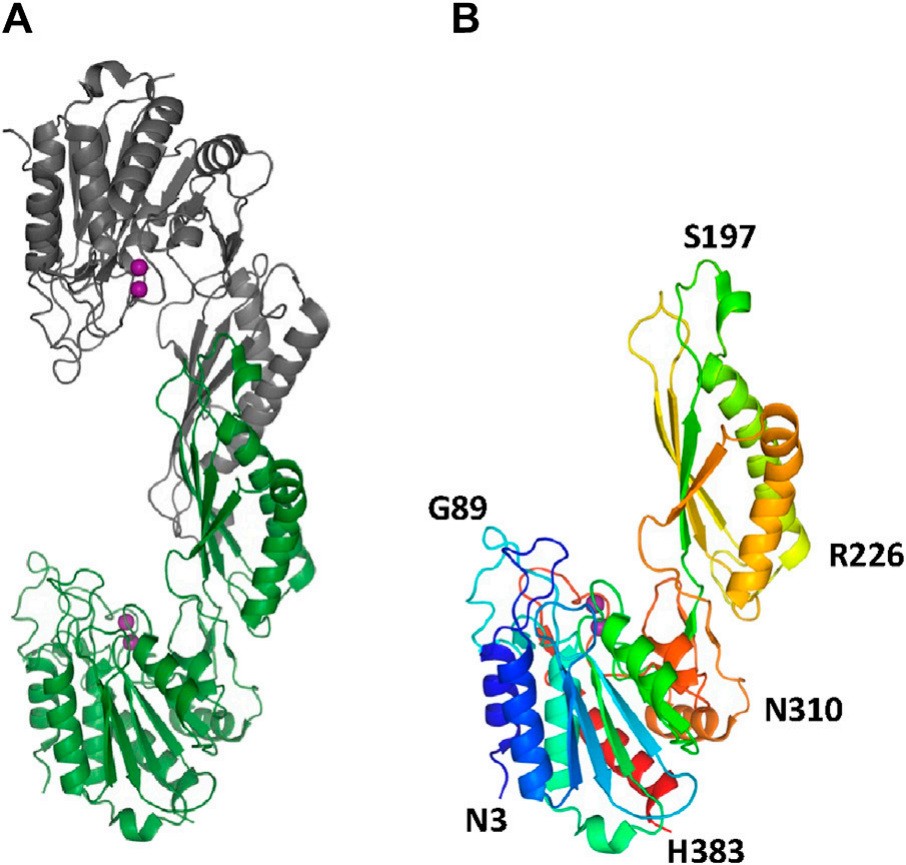
The crystal structure of *Ec*ArgE. Zinc atoms are shown as magenta spheres. **(A)** Dimeric *Ec*ArgE, **(B)** The *Ec*ArgE monomer shown as a rainbow cartoon.

**FIGURE 6:**
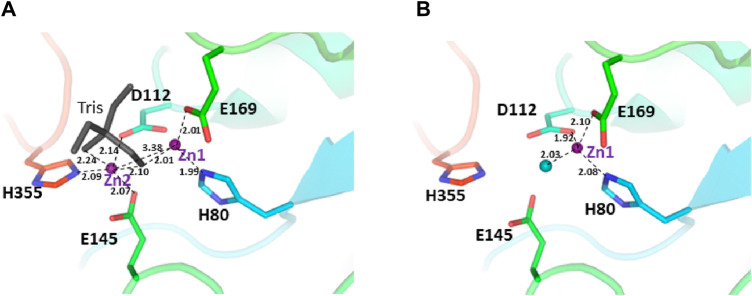
The active site of the *E. coli* ArgE protein. **(A)** di-zinc form and **(B)** mono-zinc form. The protein is shown as a rainbow cartoon with crucial residues shown as sticks. Zinc ions are shown as magenta spheres, water as a blue sphere, and the tris molecule as gray sticks. The essential hydrogen bonds are shown as dotted lines.

The two structures show a dimeric protein present in the crystals which is likely to be the biologically active form, analogous to published data on the DapE protein (Figure 5) (Nocek al., 2014). The protein monomers consist of two catalytic domains (residues 1–179 and 305–383) and a dimerization domain (residues 180–304), with a small hinge region connecting them. Protein dimer formation is achieved exclusively by the dimerization domains via hydrogen bonds between two four-stranded anti-parallel β-sheets, which together form an 8-stranded sheet. Two pairs of α-helices flanking the β-sheets on one side contribute to dimer stabilization through hydrophobic interactions. The catalytic domain is formed by a 6-stranded β-sheet hydrophobic core surrounded by 9 α-helices and two short 3-stranded β-sheets. The zinc-binding sites are located on the surface of the catalytic domain within the crescent-shaped substrate-binding cavity, and the zincs are coordinated by H80, E169, D112, E145 and H355 residues (Figure 6). The first three residues (H80, E169, D112) bind Zn1, which is present in both structures, and the last three residues (D112, E145, H355) bind Zn2, so that D112 interacts with both metal ions.

The presence of zinc ions in the structures was confirmed by anomalous and fo-fc difference electron density maps. The occupancy of the second zinc ion in the di-zinc structure was less than 100% and was estimated to be 80% based on visual inspection of the difference maps and refinement of metal positions. This feature is in very good agreement with the majority of di-zinc structures of DapE proteins available from the Protein Data Bank (PDBs 3IC1, 4H2K, and 4OP4) where the second metal ion occupancies are in the range of 50%–85%. Several di-zinc DapE structures showed full occupancy for the second zinc ion, especially those with bound ligands. Recent QM/MM calculations suggested the promiscuity of the second metal binding site in DapE enzymes (Paul and Mishra, 2021). According to the calculations, the second site can bind cobalt ions to form a Zn-Co DapE form which can outperform the Zn-Zn protein in substrate binding and catalytic efficiency. These calculations also showed that nickel and copper ions in the second position may have similar properties to the Zn-Zn pair. It has been proposed that the promiscuity of the second metal binding site is an evolutionary advantage if limited amounts of zinc ions are available. This hypothesis also explains the lower occupancy of the second zinc ion in the structures due to lower specificity and affinity.

**FIGURE 7:**
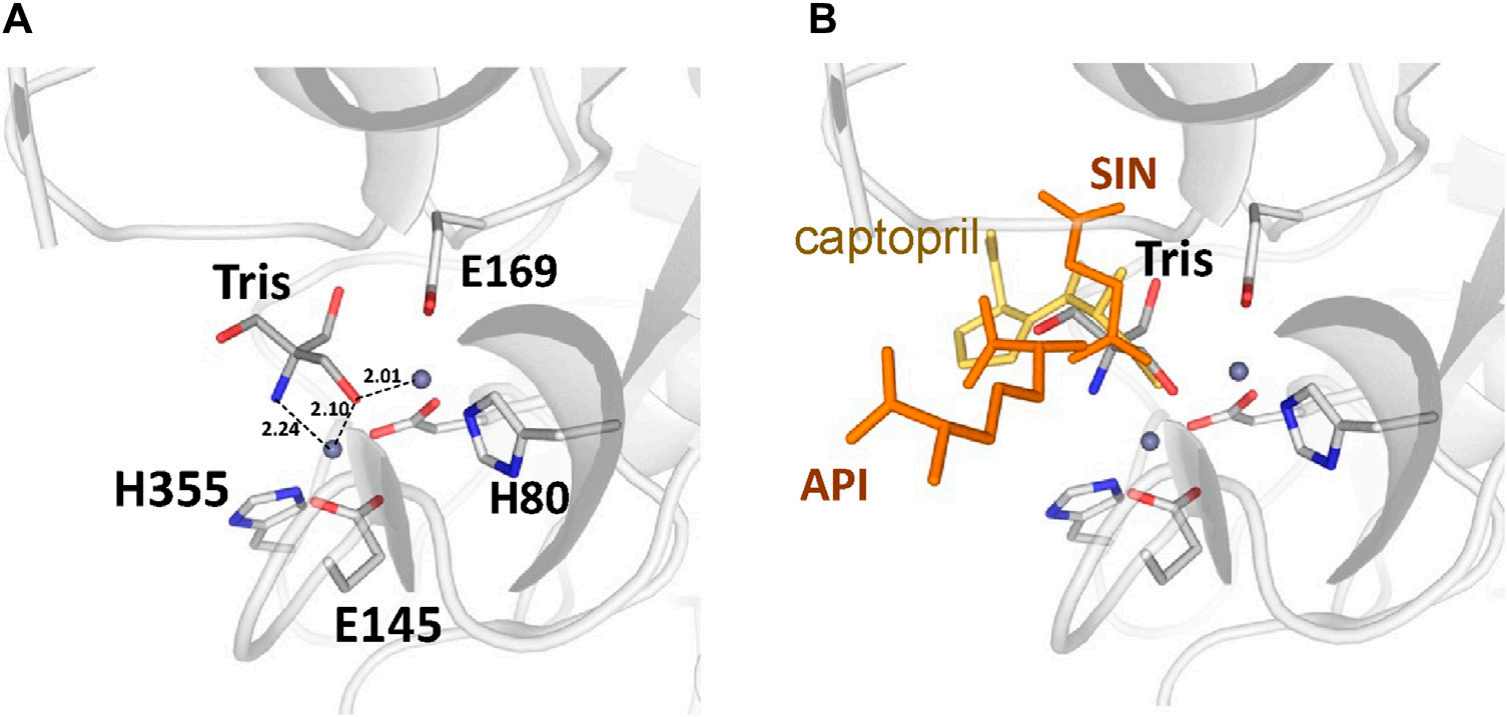
**(A)** Tris molecule in the active site of the *E. coli* ArgE di-zinc form structure (grey), **(B)** Superposition of ligands from *Neisseria meningitidis* DapE (captopril as yellow sticks, PDB 4PQA) and *Haemophilus influenzae* DapE (succinic acid (SIN) and diaminopimelic acid (API) as orange sticks, PDB 5VO3).

There are no major structural differences between the mono-zinc (PDB 7RSF) and di-zinc (PDB 8UW6) structures, either in the active site or in the overall monomer/dimer structure. There are two minor differences which are 1) a slight rotation of the dimerization domain relative to the catalytic domain, and 2) the presence of a Tris-buffer molecule bound in the active site of the di-zinc protein form. The Root Mean Square Deviation (RMSD) between the two structures as monomers is equal to 0.8 Å. When the two ArgE domains are aligned separately, the RMSD for the catalytic and dimerization domains are 0.47 and 0.40 Å, respectively. It should be noted that the structures were obtained from different crystallization conditions and represent different crystal forms as judged by differences in crystallographic symmetry, unit cell parameters, and crystal packing. The same protocol was used for the protein purification of both zinc forms. Zinc ions were introduced into the protein only from the bacterial growth medium as no zinc-containing buffers were used during protein purification. The introduction of the second zinc may be due to differences in crystallization conditions such as salt concentration and pH, but the presence of Tris buffer appears to contribute to the binding of the second zinc ion into the protein within the crystals. The Tris molecule directly interacts with five protein residues (D112, E144, E145, E169, and H355) that are involved in zinc binding and also with both zinc ions (Figure 7A). One of the Tris oxygens is positioned between the zinc ions and may stabilize the second zinc ion in the active site. This Tris oxygen mimics the captopril sulfur atom in the DapE-captopril structure (PDB 4PQA) (Starus et al., 2015) and the succinic acid oxygen in the DapE-products-bound structure (PDB 5VO3) (Nocek et al., 2018) (Figure 7B). Tris is a weak inhibitor of ArgE with 21.2% ± 0.93% inhibition at 2 mM. Upon examining the crystallization buffer, the Tris concentration is 100 mM, thus acting as an inhibitor at this concentration. It is known that Tris can inhibit zinc-containing metalloenzymes at very high concentrations, where it can interact with zincs in the active site (Hansen et al., 1993; Handing et al., 2018).

**FIGURE 8:**
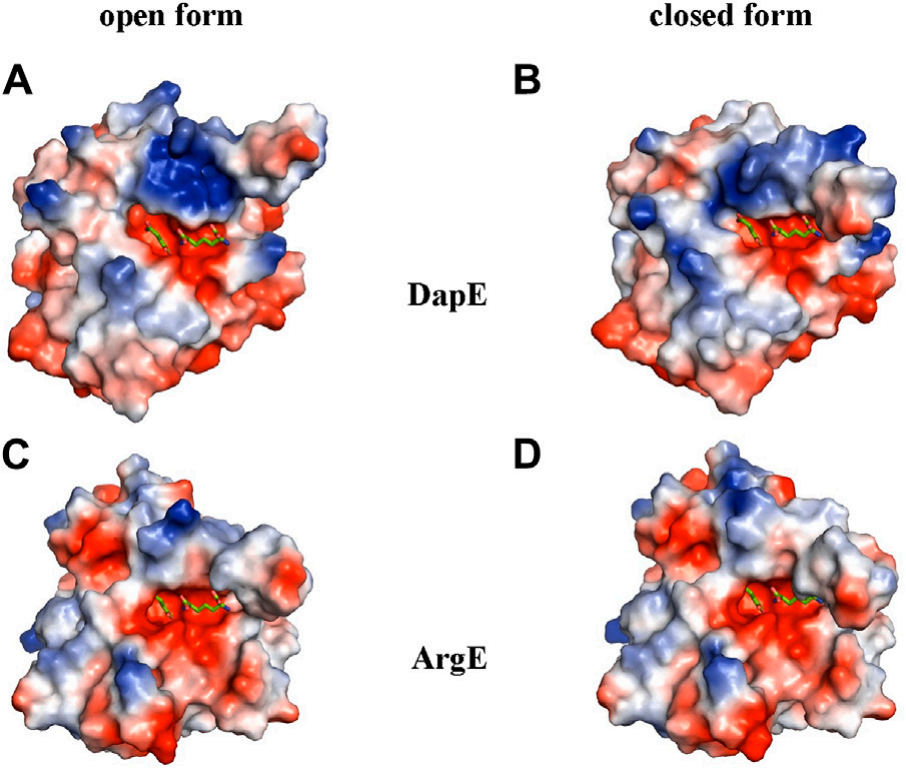
ArgE and DapE substrate-binding pockets. The DapE products (based on the *Haemophilus influenzae* DapE structure (PDB 5VO3)) are displayed as sticks over the electrostatic surface potential of the protein structures. **(A)** *H. influenzae* DapE open form (PDB 3IC1), **(B)** *H. influenzae* DapE closed form (PDB 5VO3), **(C)** *E. coli Arg*E open form (PDB 8UW6), **(D)** *E. coli Arg*E closed form generated by the rotation of the ArgE dimerization domain to match the corresponding domain of *H. influenzae* DapE closed form. The surface is colored according to charge; red is negative and blue is positive. Only single chains A of the structures with partially truncated domain dimerization were used.

**FIGURE 9:**
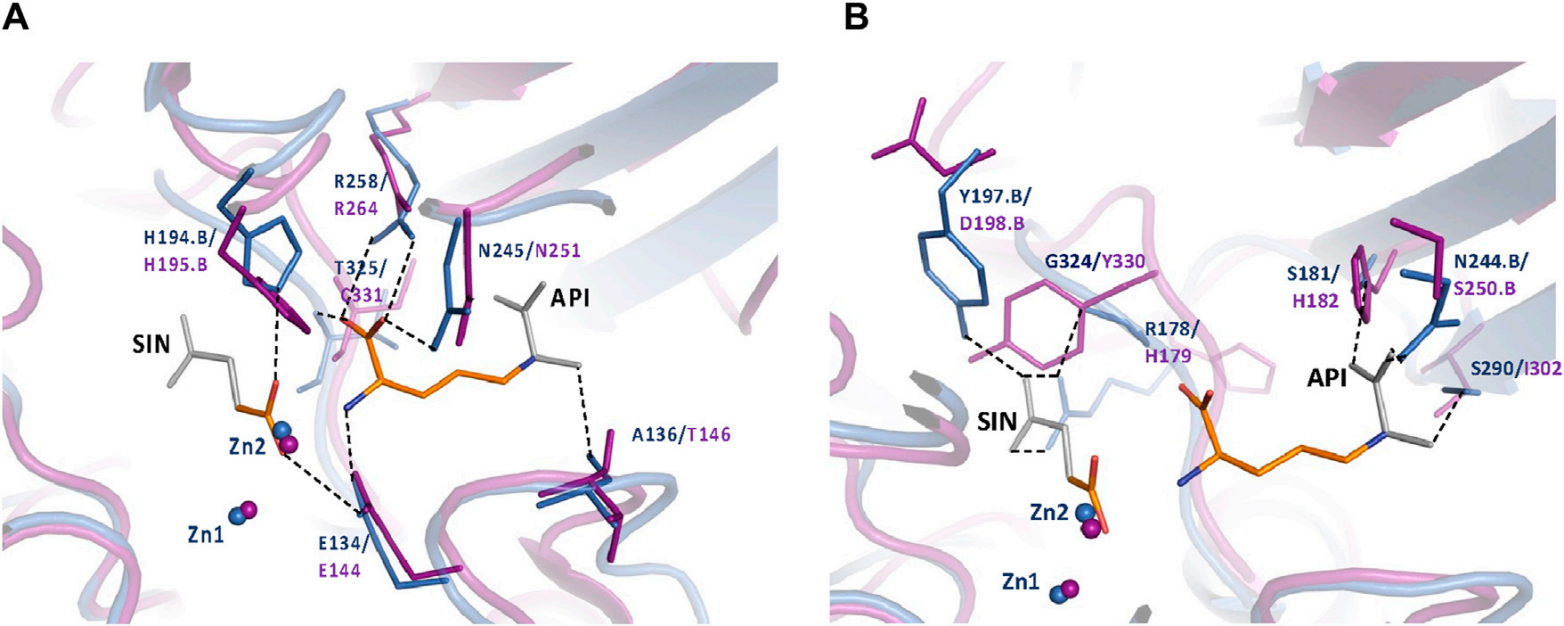
**(A)** Conserved ArgE protein-product binding. The closed form of the *E. coli* ArgE protein (in magenta) and product binding were modeled based on the closed form of *H. influenzae* DapE (in blue) (PDB 5VO3). ArgE products, acetate and L-ornithine, are shown as orange sticks. Parts of the DapE reaction products, succinic acid (SIN) and diaminopimelic acid (API), which are not present in the ArgE products are shown as gray sticks. The bonds of DapE products are shown (dashed lines) only if they interact with substrates and would be present for the ArgE protein. Residue numbers are shown in DapE/ArgE format with an added ‘B’ if the residue comes from the other protein monomer rather than the one with the observed binding site. Residues T325/C331 (located behind the API molecule) and A136/T146 bind the substrate/products through the main-chain nitrogen and oxygen, respectively. The interactions of the SIN product with the zinc ions are not shown for clarity. **(B)** DapE interactions with its products which are not conserved in the ArgE active site. The closed-form *E. coli Arg*E protein (in magenta) was superimposed on the *H. influenzae* DapE structure (in blue) (PDB 5VO3). The ArgE products, acetate and L-ornithine, are shown as orange sticks. Parts of the DapE reaction products, succinic acid (SIN) and diaminopimelic acid (API), which are not present in the ArgE products, are shown as gray sticks. Residue numbers are shown in DapE/ArgE format with an added ‘B’ if the residue comes from a protein monomer other than the one with the binding site. Bonds of DapE products only are shown as dashed lines. Interactions of the SIN product with zinc ions are not shown for clarity.

To further understand the ArgE binding pocket, we conducted modeling experiments with the ArgE substrate. The ArgE substrate binding site is located in a crescent-shaped cavity next to the zinc ions, similar to the binding cleft of the DapE active site. We used the structure of DapE in complex with the hydrolysis cleavage products, L,L-diaminopimelic acid (L,L-DAP) and succinate (PDB 5VO3)(Nocek et al., 2018) to obtain a model of the likely ArgE-substrate interactions. In the closed conformation, the residues from the dimerization domain of the complementing monomer of the dimer move closer to the pocket and interact with ligands (Nocek et al., 2018). To evaluate substrate/product interactions with ArgE, we generated a closed-form model of the ArgE protein by superimposing the ArgE catalytic and dimerization domains on the corresponding domains of the DapE dimer structure with bound products. We placed the DapE enzymatic hydrolysis products into both ArgE forms, open (PDB 8UW6) and closed-form models, based on the DapE-product structure (Figure 8). The ArgE substrate, NAO, is smaller than the DapE substrate, L,L-SDAP. Both molecules (NAO and L,L-SDAP) bind to the same amino acid residues. The hydrolysis of L,L-SDAP by DapE takes place at the active site and forms the reaction products, L,L-DAP and succinate, which correspond to the ArgE products, L-ornithine and acetate, respectively. All modeled interactions of DapE with the L,L-SDAP bound product are strictly preserved in the ArgE closed-form model with bound NAO. The DapE residues that bind L,L-SDAP are E134, A136, H194, R258, T325 (of chain A) and N245 (of chain B), and have ArgE counterparts E144, T146, H195, R264, C331 and N251, respectively (Figure 9A). The differing ArgE/DapE residues, A136/T146 and T325/C331, respectively, interact with their respective substrates through the backbone atoms of the main chain. The major differences between ArgE and DapE are located outside the NAO (corresponding to L,L-DAP) binding site at the position of two L,L-SDAP moieties, acetyl and carboxyl, which are absent in the ArgE substrate. The DapE residues that bind the formally “missing” acetyl moiety are R178, Y197 and G324, where G324 binds the ligand via main-chain nitrogens. These residues are replaced in ArgE by H179, D198, and Y330. Specifically, the G324/Y330 substitution reduces the pocket size by placing the bulky aromatic ring at a distance of 1.03 Å from the modeled succinate acetyl group, which would create a steric clash with the ArgE structure (Figure 9B; Figures 8C,D). The other side of the ArgE pocket is also changed. The main DapE residues interacting with the carboxyl group (missing in ArgE) are S181, N244 and S290 which have corresponding ArgE residues as H182, S250 and I302. The placement of H182 residue instead of the smaller S181 would be at a distance of 1.47 Å from the modeled carboxyl moiety, which would also create a clash with the closed-form ArgE pocket (Figures 8D, 9B). Interestingly, there is enough room for the carboxyl moiety in the open state of the protein as the binding pocket is significantly larger in this case (Figure 8C). Based on the similarities and differences in both active sites, we can hypothesize that the reaction mechanism of ArgE is the same as that of DapE proteins. All of the key interactions of DapE with the L,L-SDAP core would be preserved in the ArgE-substrate complex, including the bonding and placement of zinc ions. The crucial differences between the proteins are located in areas outside of the NAO/L,L-SDAP core binding pocket which could be expected due to requirements for binding different substrates.

The assumption that the two main residues limiting ArgE pocket size are H182 and Y330 should be reflected by their conservation in other ArgE proteins. A review of protein sequences from GenBank assigned as ArgE did not fully confirm this assumption showing approximately 75% residue preservation. To verify that this was not a result of annotation errors due to similarities between proteins belonging to the M20 metallopeptidase family, we ran a blastp search using the Swiss-Prot database. This database provides a minimal level of redundancy and a broad range of protein sequence homologs with divergent levels of identity. As a result (Supplementary Figure S2), we found that all of the closest homologs with a sequence identity greater than 30% and an E value lower than 2e-49 preserved residues H182 and Y330 with the one exception: *Myxococcus xanthus* ArgE (31.5% sequence identity) (Harris and Singer, 1998) was a borderline homolog with the residues replaced by tyrosine and phenylalanine, respectively, favoring aromatic residues. This outcome strongly supports the idea that residues H182 and Y330 are essential for ArgE specificity and could be used for the identification of ArgE proteins.

This is the first published report of an ArgE protein structure. Prior to this report, there were two unpublished structures in the PDB that are annotated as putative Acetyl-L-ornithine Deacetylase/ArgE proteins: a putative Acetyl-L-ornithine Deacetylase from *R. palustris* (PDB 3PFO) and a putative Zinc Peptidase from *Bacteroides thetaiotaomicron* (PDB 3^−ΔΔCT9^). The putative closed form of *Rp*ArgE is most likely caused by crystal packing and is probably a crystallization artifact. Both proteins do not have conserved residues corresponding to *E. coli* ArgE H182 and Y330. Also, an amino-acid sequence identity search between the PDB structures shows that the closest homolog of *E.coli* ArgE is *Haemophilus influenzae* DapE (26.8% amino-acid identity, PDB 3IC1 and 5VO3) (Supplementary Figure S2). The next closest sequence homologs are Acetyl-L-citrulline Deacetylase from *Xanthomonas campestris* (25.3% identity, PDB 2F7V) and a putative Zinc Peptidase from *B. thetaiotaomicron* (24.8% identity, 3^−ΔΔCT9^). The putative ArgE from *R. palustris* is the seventh closest homolog on this list. Different lists of *E. coli* ArgE homologs are obtained from the DALI server which compares proteins based on their structure. The closest structural homolog is DapE from *Enterococcus faecium* (PDB 7UOI) with a Z-score of 41.7 and rmsd = 2.3 Å. The next homologs from the DALI list are the *R. palustris* ArgE (PDB 3PFO) (Z-score = 40.5 and rmsd = 3.7 Å) and the *Acinetobacter baumannii* DapE (Kelley et al., 2024) (PDB 7T1Q) (Z-score = 38.4 and rmsd = 3.4 Å). These results from two homology searches, based on the amino-acid sequence and structural folds, suggest possible errors in protein name annotation and/or intertwining of the ArgE and DapE protein subfamilies. This makes it difficult to assign a biological function to a protein based on amino-acid sequence alone for both proteins and the whole M20 metallopeptidase family. These results suggest that biological experiments involving protein reactions are needed to confirm predictions based only on sequence and structure annotations. We believe that our reported *E. coli* ArgE structures are the only ArgE proteins currently in the PDB.

**FIGURE 10:**
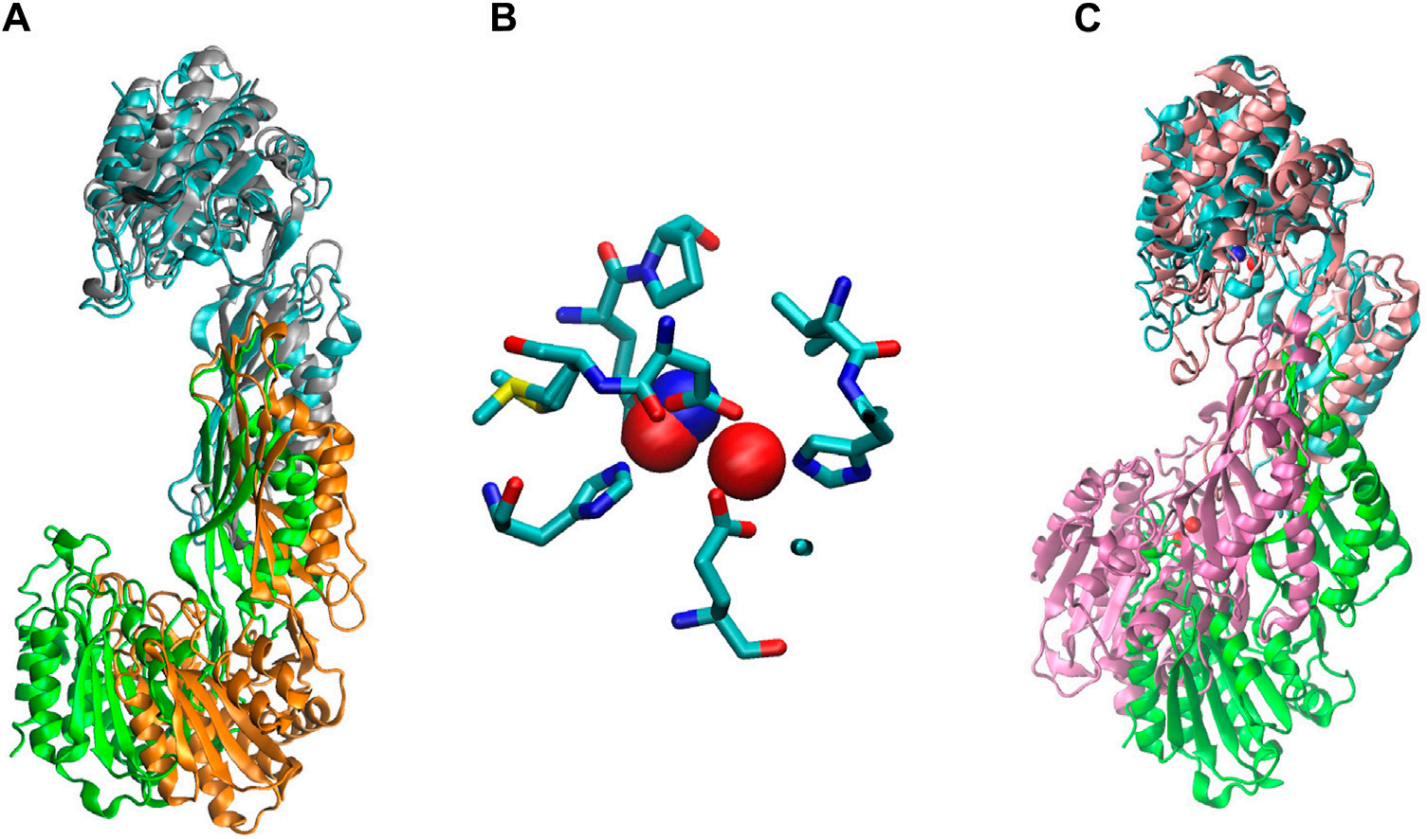
**(A)** Chain A (cyan) of *Ec*ArgE (PDB 7RSF, chain B- green) is overlayed on chain A (gray) of *Nm*DapE (PDB 5UEJ, chain B- orange), **(B)** Zinc of *Ec*ArgE (blue) is overlayed on Zinc of *Nm*DapE (red), **(C)** *Ec*ArgE (PDB 7RSF, chain B- green) chain A (cyan) is overlayed on chain A (pink) of *Rp*ArgE (PDB 3PFO, chain B- mauve).

We ran a molecular dynamics experiment with two-zinc ArgE using PDB structure 7RSF to compare our zinc-zinc distances calculated via X-ray absorption spectroscopy and X-ray crystallography. Because 7RSF is a mono-zinc structure, we needed to model the second zinc. As noted above, after overlaying chain A of *Rp*ArgE (PDB 3PFO) on *Ec*ArgE (PDB 7RSF) we observed that the 3PFO structure (Figure 10C) is in a closed conformation of ArgE, whereas 7RSF is in the open conformation. This is evident by the dynamic movement of the hinge region of the enzyme along with the 3.4 Å distance between the overlayed subunits. When we overlayed chain A of *Nm*DapE (PDB 5UEJ) on 7RSF (Figure 10A) we observed that the zincs were in almost identical locations and their Van Der Waals overlapped (Figure 10B). Therefore, we modeled the second zinc using the coordinates of *Nm*DapE, which is in the open conformation. The Zn-Zn distance determined by X-ray crystallography was observed to be 3.38 Å, nearly identical to the Zn-Zn distance of 3.39 Å determined by X-ray absorption spectroscopy. The Zn-Zn distance determined by molecular dynamics calculations indicated that the Zn-Zn distance in the most favorable conformation of *Ec*ArgE is 3.48 ± 0.06 Å , approximately 0.1 Å longer than the other two methods.

### Stable water molecules

There are five water molecules near each pair of zinc ions. Two of these water molecules are bound to the zinc ions. The fifth water is shared between the two ions and is presumably the attacking water in the hydrolysis reaction, similar to DapE (Nocek et al., 2018). All five of these water molecules in each active site were relatively fixed during the 10 ns molecular dynamics simulation. The homologous DapE enzyme has a similar set of five water molecules in its active site (PDB 3ICL) (Nocek et al., 2010). When the products are bound to DapE (PDB 5V03) (Nocek et al., 2018) one of the zinc-bound waters is replaced by the carboxylate group of the succinate. A similar situation can be anticipated for ArgE. There are 643 crystallographic waters in the AB dimer structure (374 on the A subunit and 269 on the B subunit). After 10 ns of dynamics only 34 of these waters remain in their original positions (16 on the A subunit and 18 on the B subunit). In general, stable water molecules are hydrogen-bonded to polar side chains or the backbone atoms of hydrophobic residues (Supplementary Figure S15). These stable water molecules are surrounded by protein atoms so that both hydrogen bonding and Van der Waals interactions trap them inside the protein. These water molecules are bound to the catalytic domains of ArgE rather than to the dimerization domains (Supplementary Figure S16). They are close to the active site and may play a role in stabilizing either the overall protein structure or the active site.

## Conclusion

In summary, we have designed and synthesized a new ArgE substrate, di-methyl-*N* ^*α*^-acetyl-L-ornithine (**3**), and created a new ninhydrin-based ArgE assay. Using this assay, we were able to identify inhibitors of *Ec*ArgE including captopril, which acts as a competitive inhibitor, a series of phenylboronic acids, and several benzoic acid derivatives. Among these inhibitors, 4-(diethylamino)phenylboronic acid was the most potent with an IC_50_ value of 50.1 ± 7.3 μM and we observed a potential mode of inhibition of 4-(diethylamino)phenylboronic acid via molecular docking. We have reported two new, and the only published, X-ray crystal structures of *Ec*ArgE, both in a presumed open conformation of the enzyme. We have also included X-ray Absorption Spectroscopy which is consistent with previously reported results (Tao et al., 2012). This methodology is utilized to elucidate how the substrate and inhibitors interact with the *Ec*ArgE active site, and to perform thermal shift analysis of *Ec*ArgE in the presence of the inhibitor captopril, which exhibits a K_i_ value of 35.9 μM *versus Ec*ArgE.

## Data Availability

The datasets presented in this study can be found in online repositories. The names of the repository/repositories and accession number(s) can be found below: http://www.wwpdb.org/, 7RSF, http://www.wwpdb.org/, 8UW6.
