# Corrigendum: N^α^ -acetyl-L-ornithine deacetylase from *Escherichia coli* and a ninhydrin-based assay to enable inhibitor identification

**DOI:** 10.3389/fchem.2024.1465797

**Published:** 2024-08-07

**Authors:** Emma H. Kelley, Jerzy Osipiuk, Malgorzata Korbas, Michael Endres, Alayna Bland, Victoria Ehrman, Andrzej Joachimiak, Kenneth W. Olsen, Daniel P. Becker

**Affiliations:** ^1^ Department of Chemistry and Biochemistry, Loyola University Chicago, Chicago, IL, United States; ^2^ Structural Biology Center, Argonne National Laboratory, X-ray Science Division, Lemont, IL, United States; ^3^ eBERlight, Argonne National Laboratory, X-ray Science Division, Lemont, IL, United States; ^4^ Center for Structural Biology of Infectious Diseases, Consortium for Advanced Science and Engineering, University of Chicago, Chicago, IL, United States; ^5^ Canadian Light Source, Saskatoon, SK, Canada; ^6^ Department of Biochemistry and Molecular Biology, University of Chicago, Chicago, IL, United States

**Keywords:** ArgE, ninhydrin, *Escherichia coli*, enzyme inhibition, X-ray crystal structure

## Abstract

**Figure:**
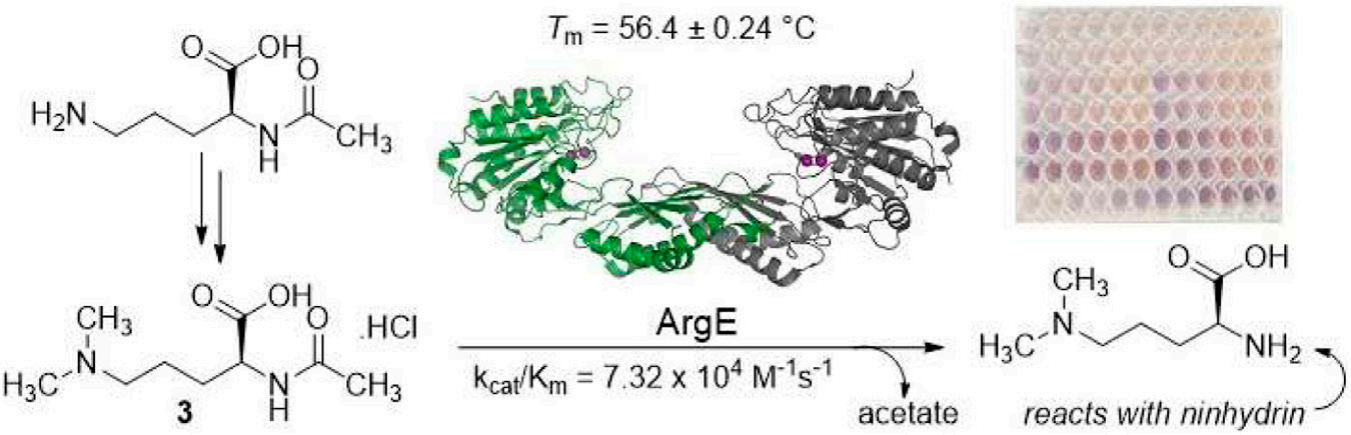

In the published article, there was an error in the **Graphical abstract** as published. A figure from the manuscript was substituted for the intended **Graphical abstract** image. The correct **Graphical abstract** appears below.

The authors apologize for this error and state that this does not change the scientific conclusions of the article in any way. The original article has been updated.

