# Supplementary material for: N α -acetyl-L-ornithine deacetylase from Escherichia coli and a ninhydrin-based assay to enable inhibitor identification
Source: Front Chem. 2024 Jul 11;12:1415644. doi: 10.3389/fchem.2024.1415644 (PMC11270798; doi:10.3389/fchem.2024.1415644)
Supplement: Supplementary file 1 [file DataSheet1.pdf]

Supplemental Information

N^{α}-acetyl-L-ornithine Deacetylase from *Escherichia coli* and a Ninhydrin-based Assay to enable Inhibitor Identification

Emma H. Kelley[1], Jerzy Osipiuk[2, 3, 4], Malgorzata Korbas[6], Michael Endres[4], Alayna Bland[1], Victoria Ehrman[1], Andrzej Joachimiak[2, 4, 5], Kenneth W. Olsen[1], and Daniel P. Becker[1*]

[1]Department of Chemistry and Biochemistry, Loyola University Chicago, 1068 West Sheridan Road, Chicago, IL 60660, USA.

[2] Structural Biology Center, Argonne National Laboratory, X-ray Science Division, 9700 S. Cass Ave., Lemont, IL, USA

[3]eBERlight, Argonne National Laboratory, X-ray Science Division, 9700 S. Cass Ave., Lemont, IL, USA

[4]Center for Structural Biology of Infectious Diseases, Consortium for Advanced Science and Engineering, University of Chicago, Chicago, IL, USA.

[5]Department of Biochemistry and Molecular Biology, University of Chicago, Chicago, IL, USA

[6]Canadian Light Source, 44 Innovation Blvd, Saskatoon, SK S7N 2V3, Canada.

Clustal Results:

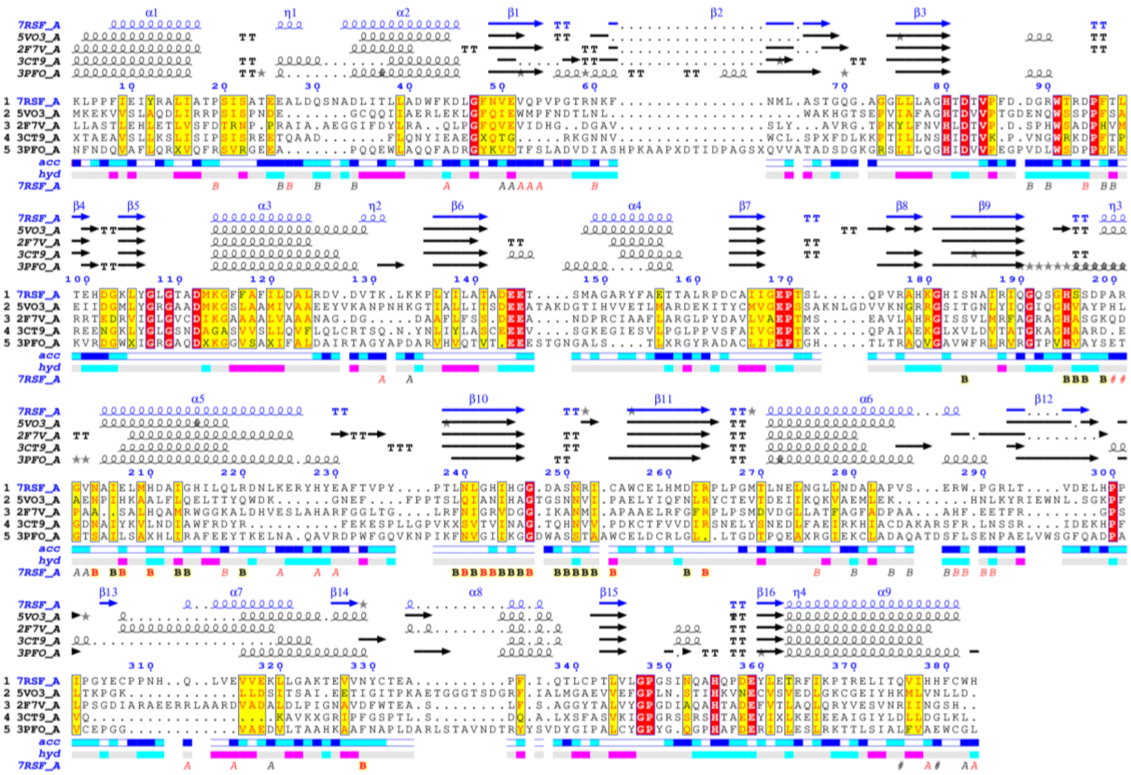

Figure S1: Sequence alignment of *E. coli* ArgE structural homologs based on amino-acid sequence homology. Three top homologs plus *R. palustris* ArgE structure (ranked as 9th best) were used. The protein secondary structures are shown above the alignments as α# for α-helices, β# -β-strands, η – 3_{10} helices, TT – β-turns. Solvent accessibility is rendered by a first bar below the sequence (blue is accessible, cyan is intermediate, white is buried) and hydropathy by a second bar below (pink is hydrophobic, white is neutral, cyan is hydrophilic). The following structures are shown: *E.coli* ArgE mono-zinc form (PDB 7RSF), *Haemophilus influenzae* DapE (5VO3), *Xanthomonas campestris* acetylcitrulline deacetylase (2F7V) and *Bacteroides thetaiotaomicron* putative zinc peptidase (3CT9) and *Rhodopseudomonas palustris* ArgE (3PFP). This figure was prepared using the ENDscript program.[Gouet, P., Robert, X., and Courcelle, E. (2003). ESPript/ENDscript: extracting and rendering sequence and 3D information from atomic structures of proteins. *Nucleic Acids Res.* 31, 3320-3323]

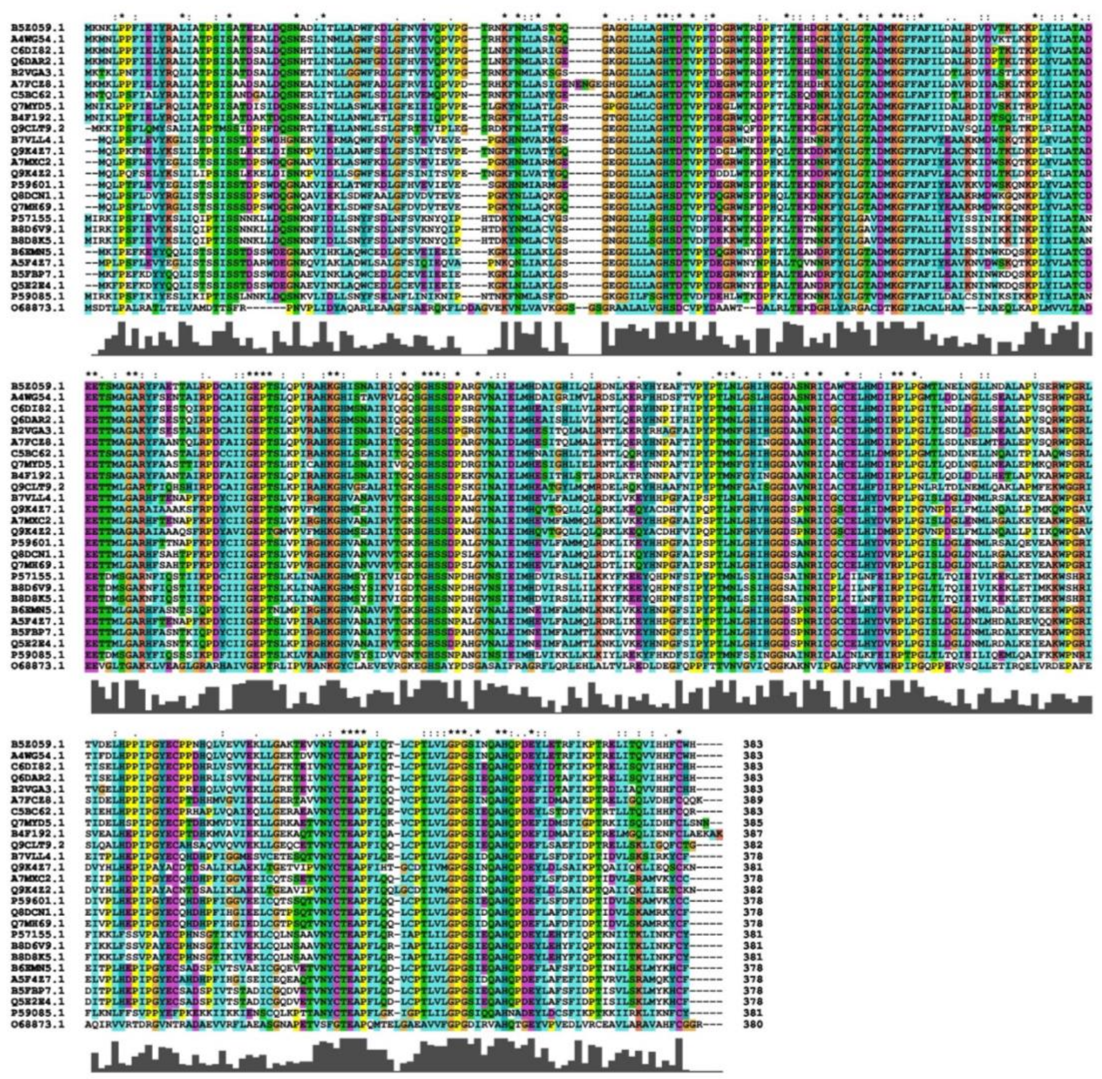

Figure S2: ClustalX alignment of amino-acid sequences from blastp search for homologs of *E. coli* ArgE (top sequence) using Swiss-Prot database. Only sequences in range of 30-90 % of identity were used. *E. coli* ArgE H182 and Y330 residues are marked with red asterisks.

X-ray Absorption Spectroscopy Study

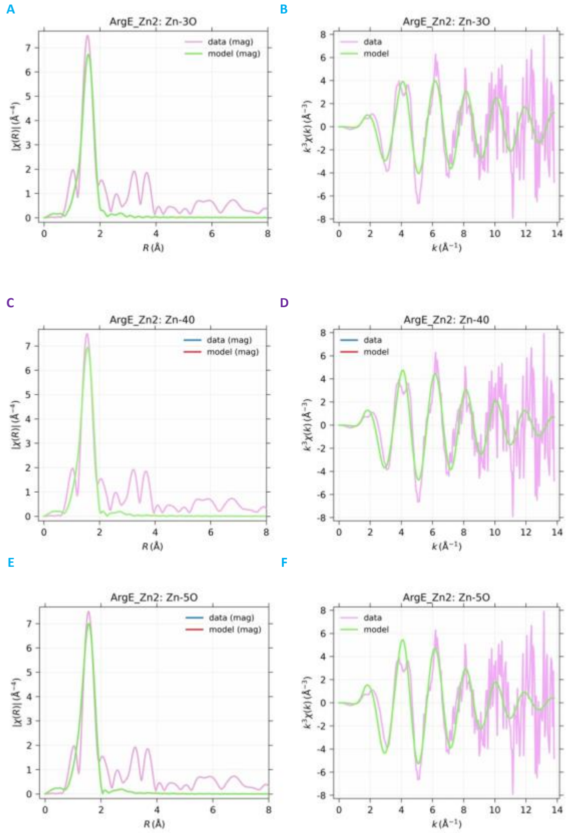

Figure S3: Zn K-edge k^3-weighted EXAFS (**B, D,** and **F**) and the corresponding Fourier transform (**A, C,** and **E**) of *Ec*ArgE after Zn dialysis (Zn2). Pink plot shows the experimental data, green is the fitted first coordination shell data. Blue lettering signifies improved fit. The best fitted Zn coordination is shown in **Table S1**.

Table S1: EXAFS First Shell Fit Data of *Ec*ArgE Zn2.

Atom	N	r (Å)	s^2 (Å^2)	reduced c2
O	3	1.97(2)	0.004(1)	8.99
O	4	1.98(2)	0.006(1)	8.20
O	5	1.98(2)	0.008(1)	10.23

The following table is the EXAFS fitted results, where N is the coordination number, r is the mean distance from the absorbing Zn atom and s^2 is the Debye-Waller factor. The quality of the fit is measured by the reduced c^2**.** The numbers in the parentheses are the errors of the last digit. The E_0 offset was refined as 11 eV +/- 4 eV for all shown fits. The best fit (lowest reduced c^2) was obtained with 4 O atoms at 1.98 Å. The EXAFS analysis is not able to distinguish between O and N atoms, therefore the first shell could be composed of a mixture of O/N atoms.

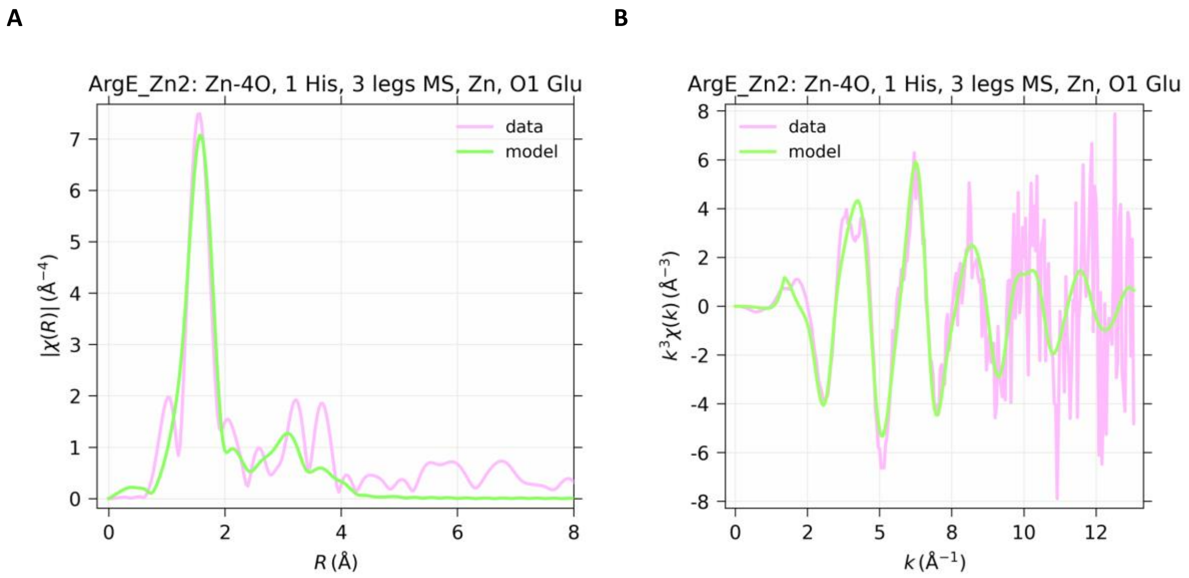

Figure S4: Zn K-edge k^3-weighted EXAFS (**B**) and the corresponding Fourier transform (**A**) of *Ec*ArgE after Zn dialysis (Zn2). Pink plot shows the experimental data, green is the fitted data using the Zn coordination model shown in **Table S2**.

Table S2: Average Zn Coordination in *Ec*ArgE Zn2 Derived from EXAFS Data Analysis.

Ligand	Atom	N	r (Å)	s^2 (Å^2)	reduced c2
H_2O/Asp/Glu	O	3	1.98(1)[a]	0.0060(7)[b]	3.75
His	N	1	1.98(1)[a]	0.0060(7)[b]	
	C1	2	3.02(1)	0.007(6)[c]	
	N2	1	4.18(1)	0.007(6)[c]	
	C2	1	4.28 (1)	0.007(6)[c]	
Glu	O1	1	2.48(5)	0.0060(7)[b]	
Zn	Zn	1	3.39(4)	0.010(4)	

The following table is the EXAFS fitted results, where N is the coordination number, r is the mean distance from the absorbing Zn atom and s^2 is the Debye-Waller factor. Only single scattering paths' parameters are shown here. The quality of the fit is measured by the reduced c^2. The numbers in the parentheses are the errors of the last digit. The E_0 offset was fitted as 11 eV +/- 3 eV. The histidine residue is represented by an imidazole ring (N, C1, C2, N2).

[a] first shell distance to O/N atoms was refined together to not overinterpret the data

[b] Debye-Waller factors were refined together

[c] Debye-Waller factors for C1, C2 and N2 atoms of His residue were refined together

Table S3: X-Ray Crystal structure data processing and refinement statistics.

	mono-zinc form	di-zinc-form
**Data processing** [a]		
Space group	$P222_1$	$P2_1$
Cell dimensions *a, b, c* (Å), *α, β, γ* (°)	47, 71, 290, 90, 90, 90	52, 126, 123, 90, 90.9, 90
Resolution range (Å)	46.2-2.13 (2.18–2.13)	48.5-1.80 (1.83-1.80)
Unique reflections	54,637 (2,396)	144,787 (7,089)
Completeness (%)	98.8 (90.5)	97.6 (96.5)
Mean I/sigma(I)	15.7 (1.02)	14.8 (1.24)
R_{merge}	0.120 (1.01)	0.119 (1.00)
R_{meas}	0.133 (1.19)	0.132 (1.11)
R_{pim}	0.057 (0.604)	0.057 (0.478)
CC1/2[c]	0.986 (0.513)	0.995 (0.462)
Redundancy	4.9 (3.1)	5.4 (5.3)
Wilson B-factor (Å^2)	34.5	19.6
**Refinement**		
Resolution range (Å)	46.2 - 2.13	48.5-1.80
Reflections work/test	51,807 / 2776	137,493 / 7260
R_{work}/R_{free}	0.188 / 0.234	0.163 / 0.203
RMSD (bonds) (Å)	0.009	0.009
RMSD (angles) (°)	1.62	1.48
**Number of atoms**		
Protein chains	2	4
Protein	5,977	12,121
Zinc	2	10
Ligands	13	81
Water	155	1,210

**B-factors**		
Average B-factor (Å^2)	49.8	27.7
Protein	49.9	27.0
Zinc	38.5	22.3
Ligands	72.1	31.9
Water	46.3	34.8
**Molprobity validation** [d]		
Ramachandran outliers (%)	0.00	0
Ramachandran favored (%)	97.2	98.3
Rotamer outliers (%)	3.9	1.9
Clashscore	5.2	2.1
MolProbity score	1.87	1.20
PDB id	**7RSF**	**8UW6**

[a] Values in parentheses correspond to the highest resolution shell.
[b] R-merge = $\Sigma h \Sigma j|I_{hj} - \langle I_h \rangle| / \Sigma h \Sigma j I_{hj}$, where I_{hj} is the intensity of observation j of reflection h.
[c] As defined by Karplus and Diederichs.[Karplus, P. A.; Diederichs, K. Linking crystallographic model and data quality. Science 2012, 336, 1030-1033.]
[d] As defined by Molprobity.[Davis, I. W.; Murray, L. W.; Richardson, J. S.; Richardson, D. C. MOLPROBITY: structure validation and all-atom contact analysis for nucleic acids and their complexes. Nucleic Acids Res. 2004, 32, W615-W619.]

*Spectral data for N^5,N^5-di-methyl N^α-acetyl-L-ornithine (**3**)*

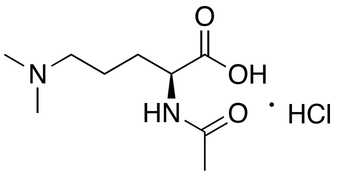

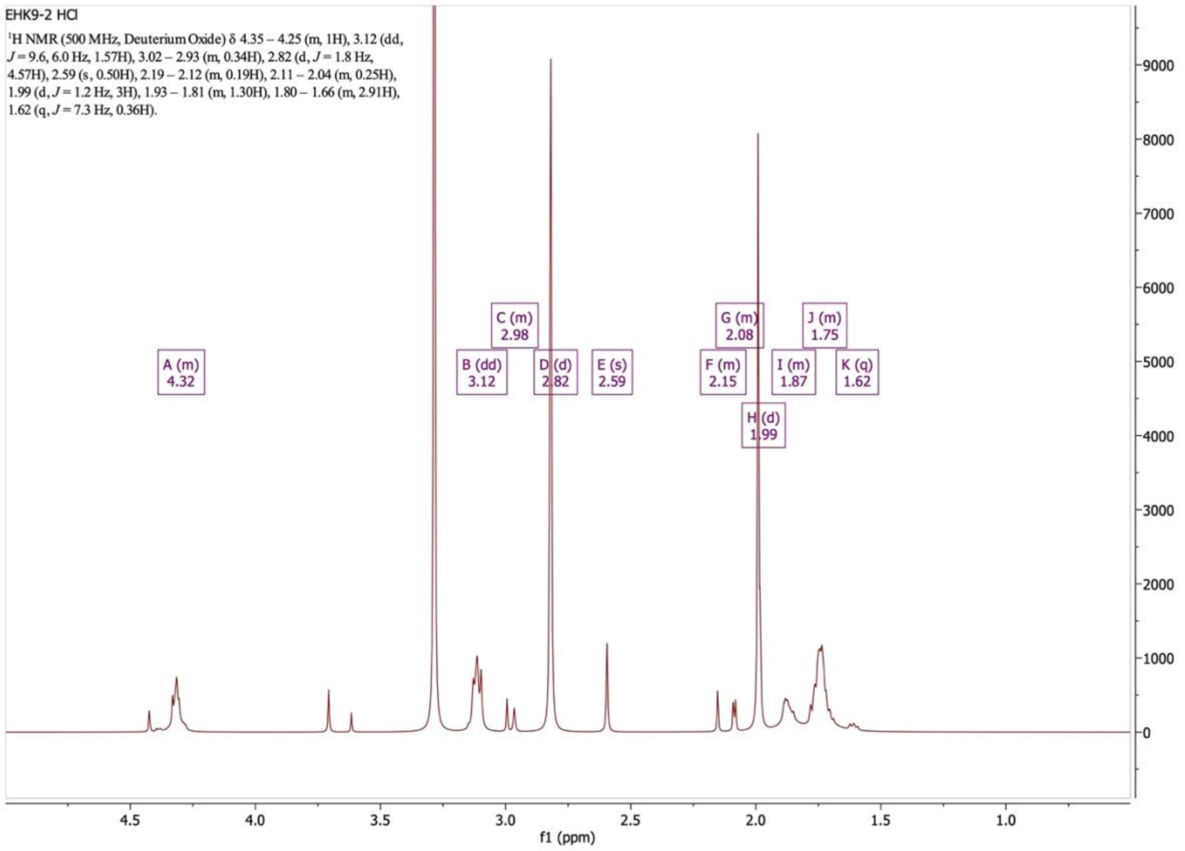

Figure S5: ^{1}H NMR (500 MHz D_2O) of N^5,N^5-dimethyl N^α-acetyl-L-ornithine (**3**).

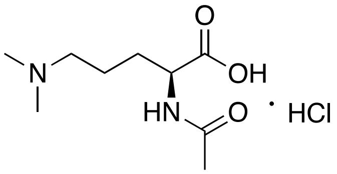

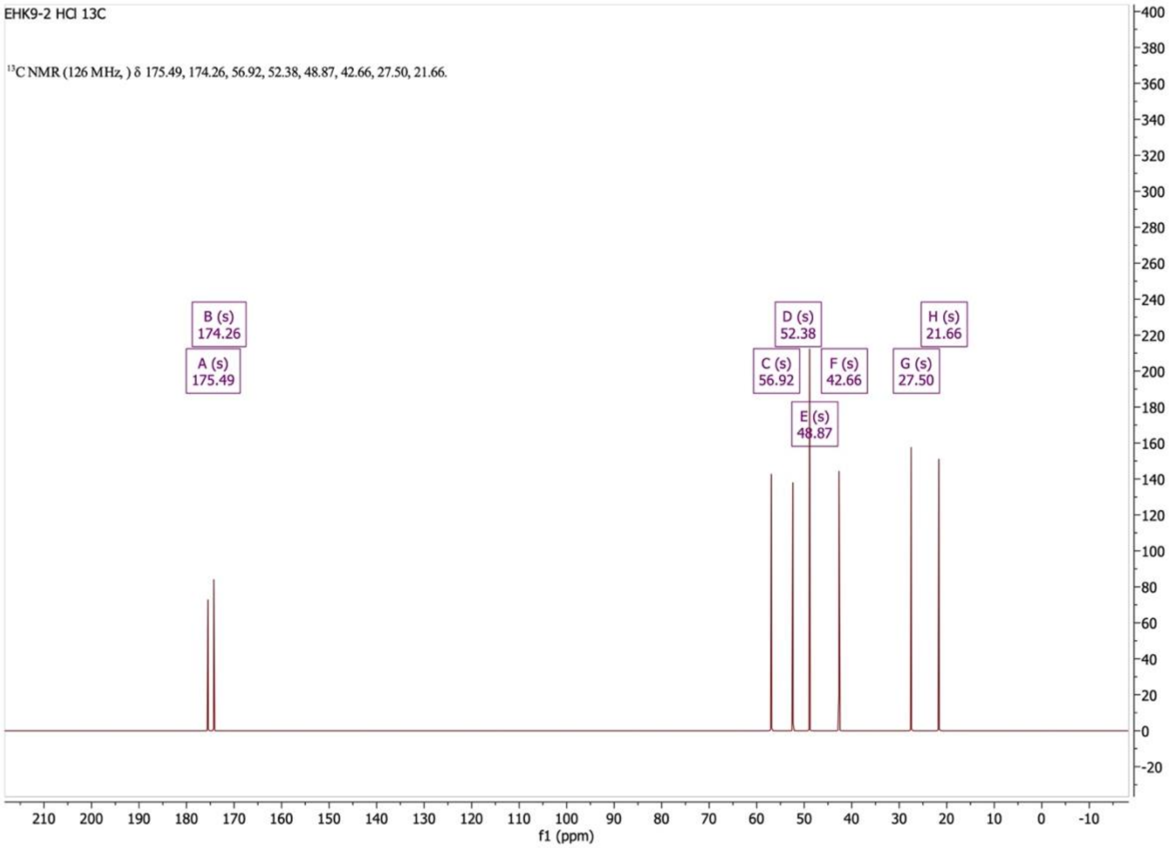

Figure S6: ^{13}C NMR (126 MHz, Methanol-*d*) of N^5,N^5-dimethyl N^α-acetyl-L-ornithine (**3**).

Assay for Inhibition of EcArgE

Ninhydrin-based assay Development

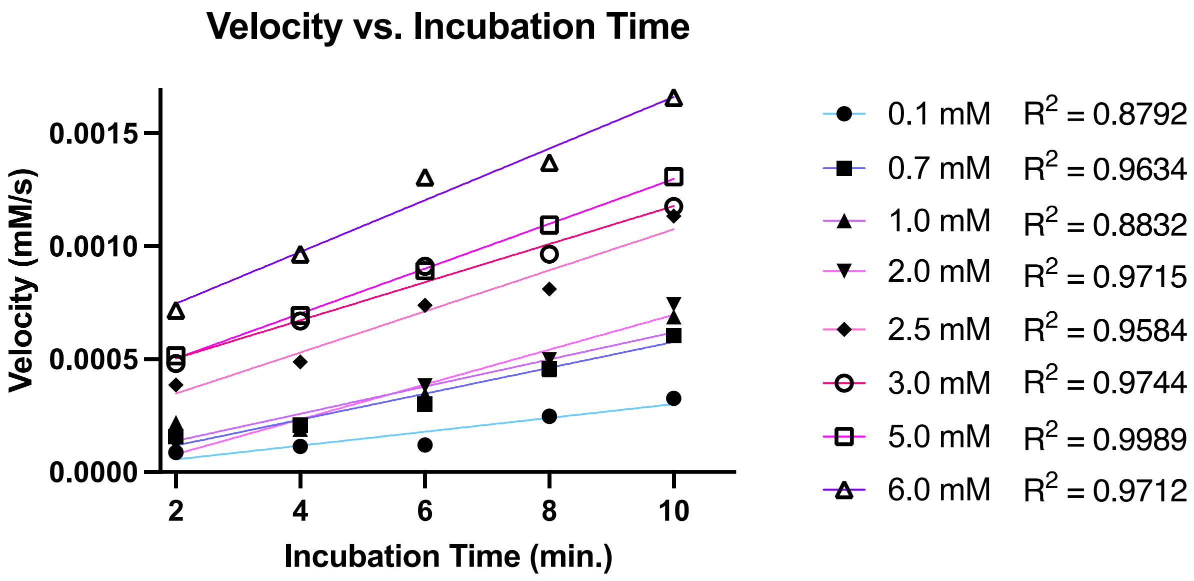

Figure S7: Graph of all substrate concentrations (mM) with velocities (mM/s) at varying incubation time (min.).

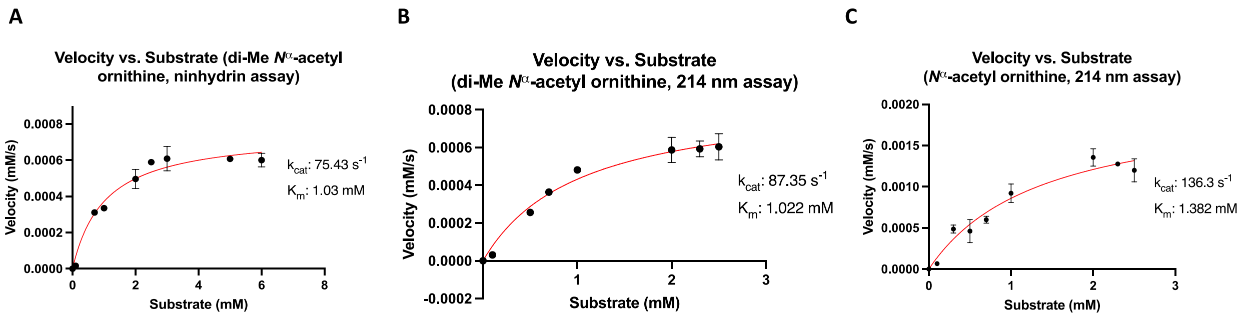

Figure S8: A) k_{cat}/K_m Graph of *Ec*ArgE in the ninhydrin-based assay with N^5,N^5-dimethyl N^{α}-acetyl-L-ornithine (**3**), **B)** k_{cat}/K_m Graph *Ec*ArgE in the 214 nm assay with di-Me N^{α}-acetyl ornithine. **C)** k_{cat}/K_m Graph *Ec*ArgE in the 214 nm assay with N^{α}-acetyl ornithine.

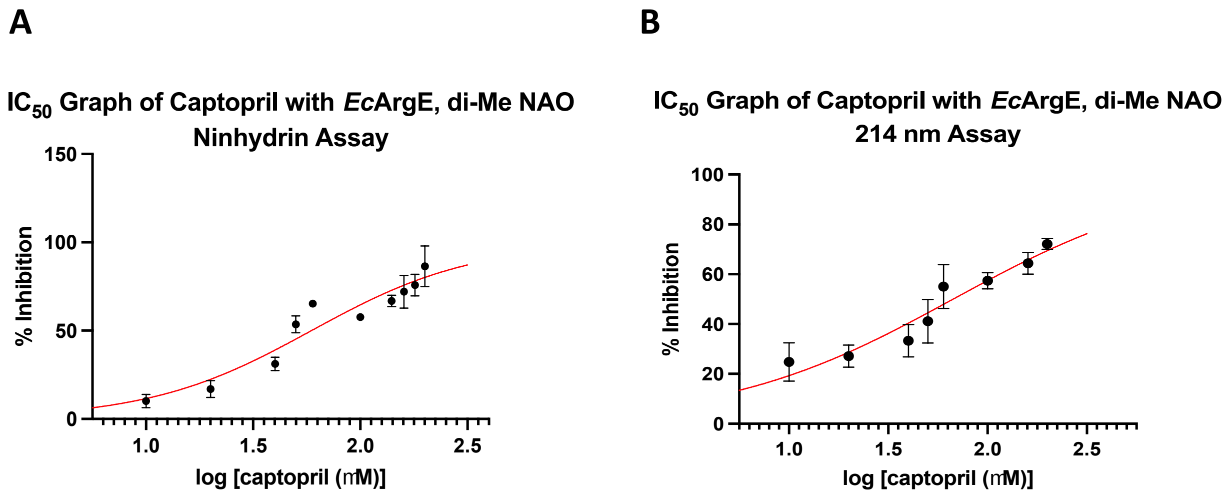

Figure S9: A) IC_{50} Graph of Captopril with *Ec*ArgE in the ninhydrin-based assay, **B)** IC_{50} Graph of Captopril with *Ec*ArgE in the 214 nm assay.

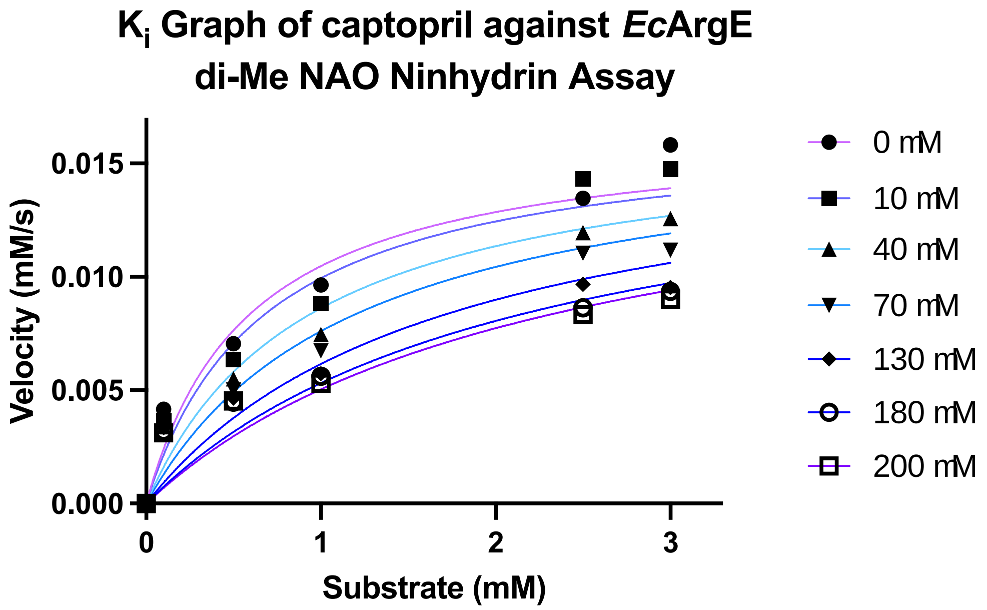

Figure S10: K_i Graph of Captopril with *Ec*ArgE in the ninhydrin-based assay.

IC_{50} Graphs of Phenylboronic Acids as Inhibitors of ArgE

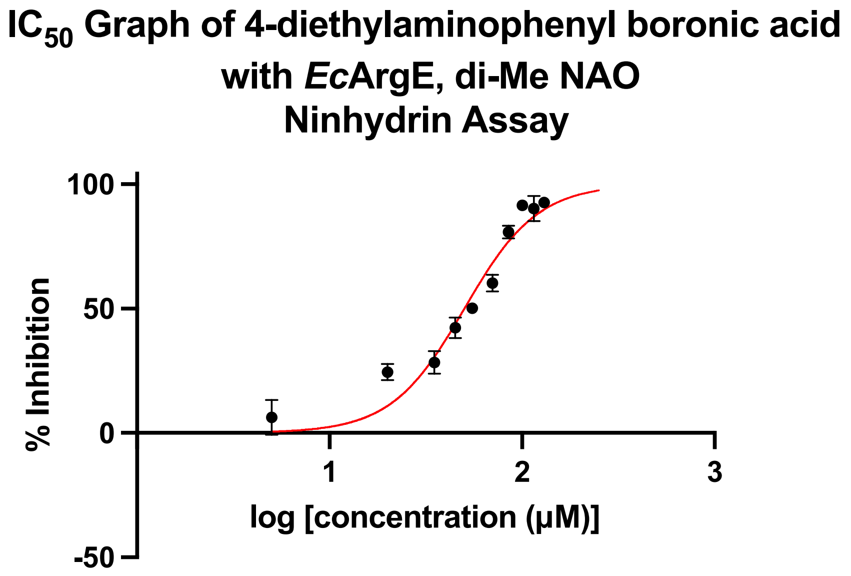

Figure S11: IC_{50} Graph of 4-diethylaminophenyl boronic acid with *Ec*ArgE.

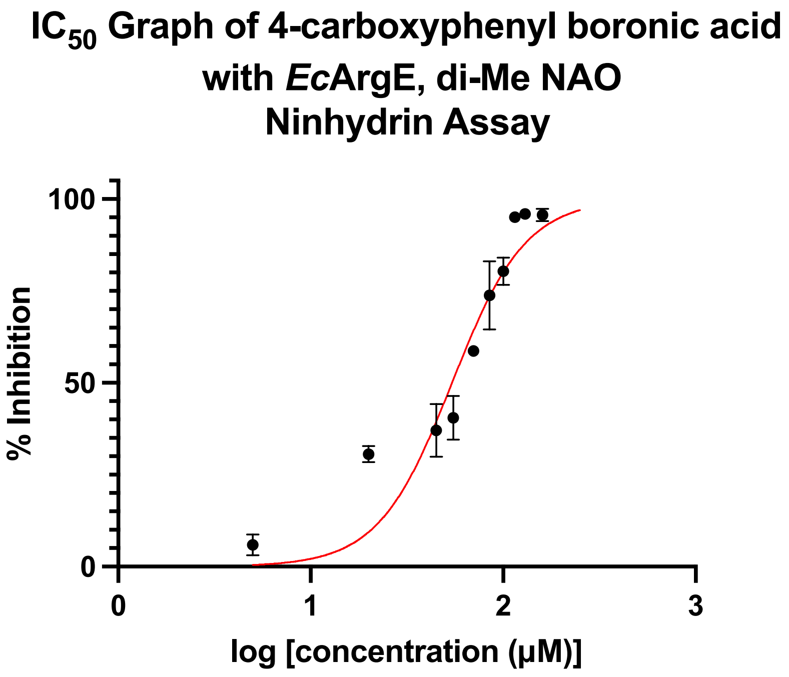

Figure S12: IC_{50} Graph of 4-carboxyphenyl boronic acid with *Ec*ArgE.

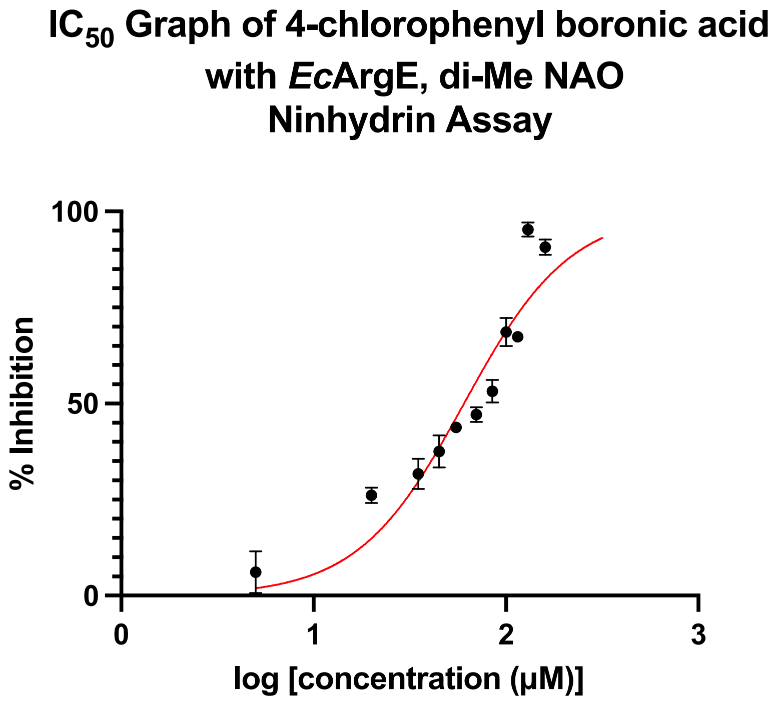

Figure S13: IC_{50} Graph of 4-chlorophenyl boronic acid with *Ec*ArgE.

Thermal Shift Assay Results

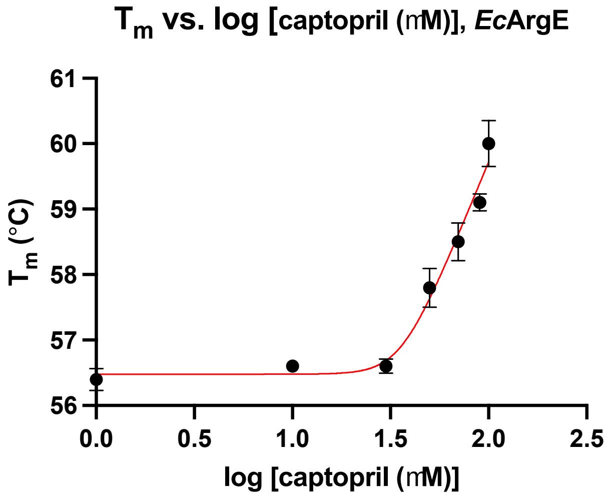

Figure S14: Thermal Shift Assay Graph of T_m vs. log [captopril (μM)]: *Ec*ArgE.

Stable Water Molecules

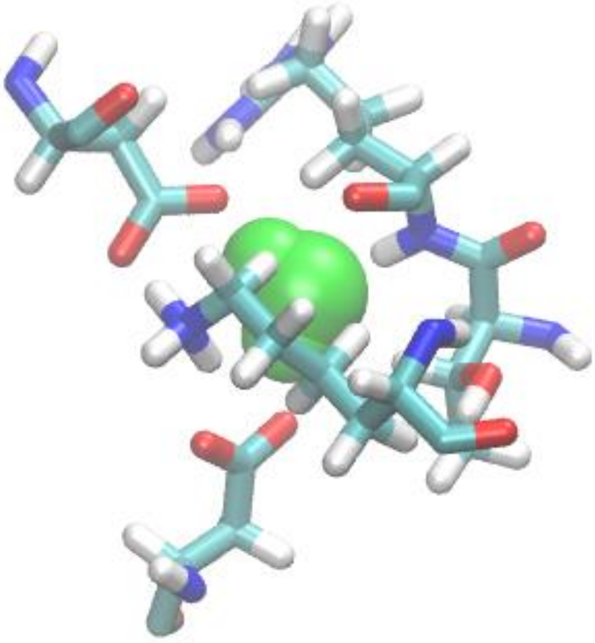

Figure S15: A stable water molecule (lime) surrounded primarily by hydrogen-binding sidechains (Chain A and residues Asp 28, Asp 143, Thr 58, Arg 59 and Lys 61).

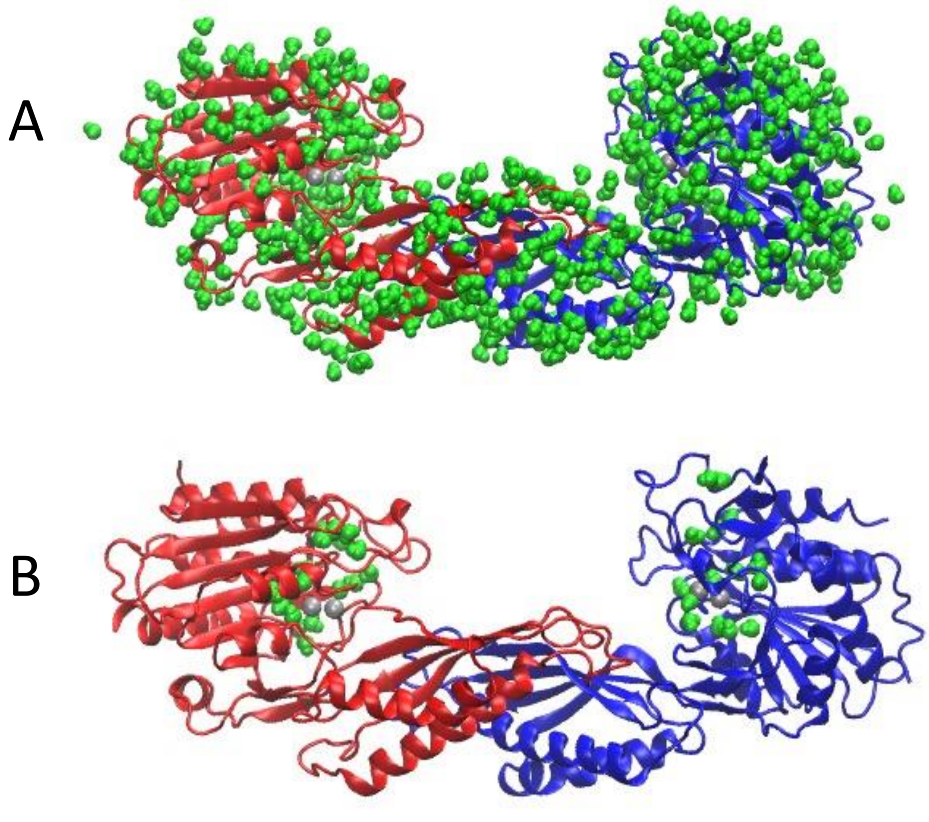

Figure S16: Comparison of waters (lime) bound to *Ec*ArgE (PDB 8UW6) before (**A**) and after (**B**) 10 ns of molecular dynamics simulation.
